# Analysis of prognostic factors for survival after surgery for gallbladder cancer based on a Bayesian network

**DOI:** 10.1038/s41598-017-00491-3

**Published:** 2017-03-22

**Authors:** Zhi-qiang Cai, Peng Guo, Shu-bin Si, Zhi-min Geng, Chen Chen, Long-long Cong

**Affiliations:** 1 Department of Industrial Engineering, School of Mechanical Engineering, Northwestern Polytechnical University, Xi’an 710072, Shaanxi, China; 2 Department of Hepatobiliary Surgery, First Affiliated Hospital, Xi’an Jiaotong University, Xi’an 710061, Shaanxi, China

## Abstract

The factors underlying prognosis for gallbladder cancer (GBC) remain unclear. This study combines the Bayesian network (BN) with importance measures to identify the key factors that influence GBC patient survival time. A dataset of 366 patients who underwent surgical treatment for GBC was employed to establish and test a BN model using BayesiaLab software. A tree-augmented naïve Bayes method was also used to mine relationships between factors. Composite importance measures were applied to rank the influence of factors on survival time. The accuracy of BN model was 81.15%. For patients with long survival time (>6 months), the true-positive rate of the model was 77.78% and the false-positive rate was 15.25%. According to the built BN model, the sex, age, and pathological type were independent factors for survival of GBC patients. The N stage, liver infiltration, T stage, M stage, and surgical type were dependent variables for survival time prediction. Surgical type and TNM stages were identified as the most significant factors for the prognosis of GBC based on the analysis results of importance measures.

## Introduction

Gallbladder cancer (GBC) is the most common malignant tumour of the biliary tract worldwide^1^. It is also the most aggressive cancer of the biliary tract with the shortest median survival from the time of diagnosis^2^. The only option for a complete cure is surgical resection. However, currently only 10% of GBC patients are candidates for surgery with a curative intent. The current international guidelines may not suit for all regions as the difference of financial resources, cultural attitudes and environmental factors. Therefore, it is of vital importance to identify practical key factors that affect the survival of patients with GBC to support the prediction of survival time and decisions regarding therapy.

Data-based statistical methods have been extensively applied to the analysis of prognostic factors for GBC patient survival^3, 4^. These studies have examined prognostic factors such as T stage, patient age, surgical type, and recurrence using statistical analyses of clinical data. However, these studies describe the separate impacts of single factors associated with prognosis and have neglected the joint influence of multiple factors. The roles of interactions or mutual influences among these factors are not yet clearly understood, so an effective modelling method is required to explore and represent the relationships among these factors.

Recent studies have analysed medical data using artificial intelligence to support specialists in the course of clinical stage, decision-making, and prognosis prediction. Wang *et al.* ^5^ developed a nomogram based on a web browser using a parametric survival model from the Surveillance, Epidemiology and End Results-Medicare database to predict which gallbladder patients may benefit from adjuvant chemoradiotherapy. Additionally, Wang *et al.* ^6^ put forward a multivariate Cox proportional hazards model to enable individualised predictions of the net survival benefit of adjuvant radiotherapy for GBC patients based on specific tumour and patient characteristics. Horgan *et al.* ^7^ undertook a systematic review and meta-analysis to determine the impact of adjuvant therapy on survival in the treatment of biliary tract cancer. Udelnow *et al.* ^8^ conducted a two-centre observational study of the accuracy of a Bayesian network (BN) for short-term outcome prediction in cholecystectomy patients. Chukwuka *et al.* ^9^ built a simple regression model to assess the variability of gallbladder contraction indices and to obtain the gastric emptying ratio. Although some methods of data mining have been developed and applied to survival prediction for patients with GBC, most methods cannot represent variables under uncertainty and ignore the cause-and-effect relationships between prognostic factors.

The BN is specialised in its representation of nonlinear and variable interactions^10^. Furthermore, importance measures are useful tools to deal with uncertainty in model prediction^11^. We have used the BN and importance measures to identify the most significant predictor of survival time for patients who have undergone hepatectomy for treatment of hepatocellular carcinoma^12^. In this paper, we used BN to construct a model that predicts prognosis for patients with GBC. Then the importance measures were used to sort these prognostic factors. The BN model, which was built based on practical medical data, could provide efficient individual prognosis and optimal treatment by considering regional health care conditions.

## Results

### General characteristics of the study population

Of the study patients, 260 (71.04%) were female and 106 (28.96%) were male. The median age at the time of surgery was 63 years. Eighty-three (22.79%) patients were positive for jaundice and 203 (55.46%) were positive for liver infiltration. Pathological analysis identified 311 (84.97%) patients with adenocarcinoma and 55 (15.03%) with non-adenocarcinoma lesions, including squamous cell carcinoma (9), neuroendocrine neoplasm (9), sarcoma (8), mucinous adenocarcinoma (11), and adenosquamous carcinoma (18). Protuberant tumours were present in 252 (68.90%) patients, while 114 (31.10%) had infiltrative lesions. The proportions of patients with lesions classified as pathological grades of well, moderate, and poor were 11.20%, 42.62%, and 46.17%, respectively. The proportions of patients with T stages of Tis, T_1a_, T_1b_, T_2_, T_3_, and T_4_ were 0.82%, 0.82%, 2.19%, 1.37%, 59.02%, and 35.79%, respectively. Meanwhile, proportions of patients with N stage lesions of grades N_0_, N_1_, and N_2_ were 28.96%, 37.16%, and 33.88%, respectively. Proportions of patients with M stage lesions of M_0_ and M_1_ were 72.40% and 27.60%, respectively. Proportions of patients requiring a total surgical time of ≤3 hours and >3 hours were 57.10% and 42.90%, respectively. Blood loss of >1000 mL occurred in 6.56% of patients.

The surgical approach used for 142 (38.80%) patients was radical surgery (R0), including 11 hepatopancreatoduodenectomy (HPD) procedures and 15 right hepatectomy or right trisegmentectomy procedures. Palliative surgical intervention was performed for the remaining cases, with 224 (61.20%) cases undergoing R1/2 resection: 72 underwent cholecystectomy, 107 cholecystectomy with biliary tract drainage, 19 biliary tract external drainage, 18 opening and closure of the abdomen, and 8 gastrointestinal anastomosis.

### Survival analysis

**Figure 1:**
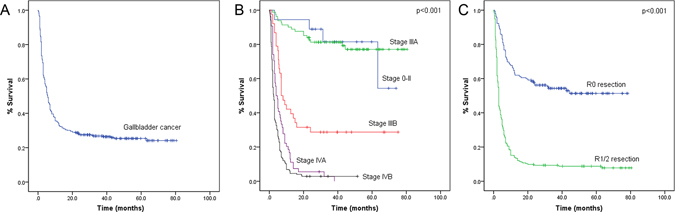
(**A**) Overall survival of GBC patients after surgery. (**B**) Survival of GBC patients with different TNM stage. (**C**) Comparison of patient survival in GBC patients after radical resection or palliative resection.

The survival curve is shown in Fig. 1. The median survival time was 5.7 months with a 95% CI of 4.9–7.0 months. The mean survival time was 24.6 months, and the 1-, 3- and 5-year overall survival rates were 34.2%, 26.8%, and 25.4%, respectively. The median survival time of patients with GBC of TNM stages 0–II and IIIA was not reached during the course of this study, with more than half of the patient cohort still alive when the study was concluded. Meanwhile, the median survival times for stage IIIB, IVA, and IVB patients were 7 months, 4 months, and 2.5 months, respectively, and the differences were statistically significant (P < 0.001). The median survival time of GBC with R1/2 resection was 3 months (P < 0.001).

### Assessment of model efficacy

**Figure 2:**
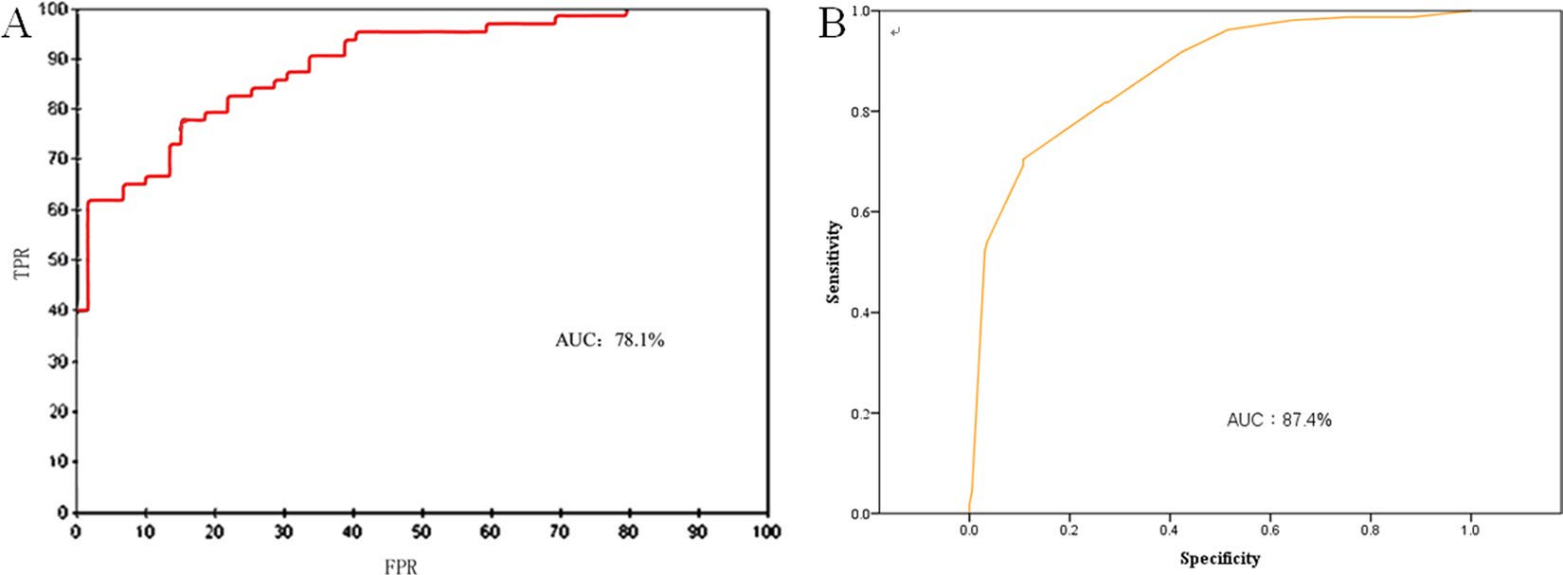
(**A**) ROC curve of survival time >6 months for BN. (**B**) ROC curve of survival time >6 months for LR.

The BN model was established after obtaining values for the required variables from 244 patient records in the training dataset to obtain a survival time. The 122 records in the testing dataset are used to test the model. The reliability and accuracy of prognosis predictions are obtained (Table 1) using confusion matrix evaluation indices with default probability threshold of 0.5. A patient was classified as having a long survival time (>6 months) when the probability was more than the threshold, otherwise the patient was classified as having a short survival time (≤6 months). The actual number of patients surviving for >6 months was 63, with 49 correctly classified—yielding a true positive rate (TPR) of 77.78%. The number of patients identified by the model was 58, and 49 of these had a survival time of >6 months, conferring a reliability of 84.48%. The above values were the predicted rates of correct classification. In the aggregate, 50 patients (≤6 months) and 49 patients (>6 months) were correctly classified, conferring a model accuracy of 81.15% (calculated as per Equation [1]). As the probability threshold varied from 0 to 1, the corresponding FPR and TPR formed the ROC curve (Fig. 2A). The area under the curve (AUC) of the receiver operating characteristics ROC for the BN model was 78.1%.

**Table 1:** Confusion matrix and reliability and accuracy of the BN model of prognosis.

	Survival time (n)	≤6 m (n = 59)	>6 m (n = 63)
Confusion matrix (n)	≤6 m (64)	182	51
	>6 m (58)	14	119
Reliability (%)	≤6 m (64)	78.12%	21.88%
	>6 m (58)	15.52%	84.48%
Accuracy (%)	≤6 m (64)	84.75%	22.22%
	>6 m (58)	15.25%	77.78%

Logistic regression (LR) analysis was implemented with the original 438 dataset in SPSS. The stepwise backward algorithm was applied with a significance threshold of 0.1. After ten steps, the final predictive model with all significant terms was obtained (Table 2). Obviously, T stage, N stage, M stage and pathological type have a significant value (p < 0.1), which can be used to generate the ROC curve (Fig. 2B). The corresponding AUC of the ROC for the LR was 87.4%.

**Table 2:** LR on survival time >6 months.

Variable	Regression coefficient	Relative risk	95% CI	P
T stage	−0.994	0.370	0.178–0.770	0.008
N stage	−1.291	0.275	0.172–0.439	<0.001
M stage	−2.157	0.116	0.041–0.327	<0.001
Pathological Type	−0.812	0.444	0.174–1.130	0.088
Constant	6.875			

### Prognostic factors ranked by importance

The importance of correlative prognostic factors was analysed according to the established BN prognostic model. First, we obtained the prior probability distribution of each factor (Table 3). The prior probability of survival time was {p(S = 0) = 0.5355, p(S = 1) = 0.4645}, and the prognostic factors that were attribute variables were described as {p(V = 0), p(V = 1), …}. Next, states of the attribute variables were modified and the posterior probability distribution of a survival time of ≤6 months was calculated. The posterior probability was determined using {p(S = 0|V = 0), p(S = 0|V = 1), …}. Finally, the importance measure of each variable was calculated using equations (2) to (8) described in the Materials and Methods. Results are shown in Table 3.

**Table 3:** Importance of prognostic factors in survival time ranking.

Prognostic factors	State	Priori probability	Posterior probability	MBM	Rank	MRAW	Rank	MFV	Rank	MRRW	Rank	MAD	Rank	MMAW	Rank	MMFV	Rank
Jaundice	0	0.7541	0.5072	0.1150	8	1.1619	6	0.0528	10	1.0558	10	0.0427	9	1.0398	9	0.0398	9
	1	0.2459	0.6222														
Liver Infiltration	0	0.4454	0.3129	0.4014	5	1.3339	4	0.4157	4	1.7114	4	0.1983	3	1.1852	3	0.1851	3
	1	0.5546	0.7143														
Pathological Type	1	0.8497	0.5113	0.1614	7	1.2562	5	0.0452	11	1.0473	11	0.0412	10	1.0385	10	0.0384	10
	2	0.1503	0.6727														
Shape	1	0.6890	0.5704	0.1122	9	1.0652	11	0.1443	7	1.1687	7	0.0480	8	1.0450	8	0.0446	8
	2	0.3110	0.4582														
Pathological Grade	1	0.1120	0.2109	0.2490	6	1.1188	7	0.3461	5	1.5293	5	0.1174	6	1.1097	6	0.1096	6
	2	0.4262	0.4808														
	3	0.4617	0.6627														
T Stage	0	0.0082	0	0.4629	3	1.0965	8	0.7679	1	4.3095	1	0.1850	5	1.1727	5	0.1727	5
	1a	0.0082	0														
	1b	0.0219	0														
	2	0.0137	0.2														
	3	0.5902	0.4213														
	4	0.3579	0.7939														
N Stage	0	0.2896	0.0755	0.4220	4	1.3585	3	0.4295	3	1.7529	3	0.2665	1	1.2489	1	0.2488	1
	1	0.3716	0.6324														
	2	0.3388	0.8226														
M Stage	0	0.7240	0.4038	0.4774	2	1.6456	1	0.2459	6	1.3262	6	0.1908	4	1.1782	4	0.1781	4
	1	0.2760	0.8812														
Age (year)	29–50	0.1366	0.5200	0.0166	13	1.0148	12	0.0162	13	1.0165	13	0.0067	13	1.0063	13	0.0063	13
	51–70	0.6503	0.5336														
	71–86	0.2131	0.5513														
Surgical Type	1	0.3880	0.2324	0.4953	1	1.3589	2	0.5660	2	2.3042	2	0.2352	2	1.2197	2	0.2196	2
	2	0.6120	0.7277														
Blood Loss	0	0.9344	0.5409	0.0826	10	1.0101	13	0.1442	8	1.1684	8	0.0101	12	1.0094	12	0.0095	12
	1	0.0656	0.4583														
Surgical Time (hour)	≤3	0.5710	0.5837	0.0562	12	1.0900	9	0.0599	9	1.0637	9	0.0550	7	1.0514	7	0.0514	7
	>3	0.4290	0.4713														
Sex	1	0.2896	0.5755	0.0563	11	1.0747	10	0.0304	12	1.0314	12	0.0232	11	1.0216	11	0.0216	11
	2	0.7104	0.5192														

### Multivariate analysis for various risk factors

After univariate analysis for the listed 13 factors with Log-rank test, 9 factors, including jaundice, liver infiltration, surgical type, T stage, N stage, M stage, pathological grade, pathological type and shape, were identified as risk factors (P < 0.05) for prognosis of GBC. Then, a multivariate analysis base on Cox regression was performed to determine which univariate prognostic relationships were independent predictive factors. The results showed that the surgical type, N stage, M stage and pathological grade were independent risk factors (p < 0.05) for prognosis of GBC (Table 4).

**Table 4:** Results of Cox multivariate regression analysis.

Variable	Regression coefficient	Relative risk	95% CI	P
Jaundice	0.182	1.200	0.834–1.727	0.325
Liver Infiltration	0.235	1.265	0.809–1.980	0.303
Surgical Type	0.638	1.893	1.243–2.882	0.003
T stage	0.144	1.154	0.744–1.791	0.522
N stage	0.619	1.856	1.466–2.350	<0.001
M stage	0.784	2.190	1.475–3.250	<0.001
Pathological Grade	0.335	1.398	1.048–1.864	0.023
Pathological Type	0.232	1.261	0.835–1.906	0.271
Shape	−0.027	0.974	0.661–1.435	0.893

## Discussion

In this study, we used a BN in combination with importance theory to identify the key factors underlying GBC patient prognosis under uncertainty. The BN model was used to predict patient survival time using data gathered from patients treated at the First Affiliated Hospital of Xi’an Jiaotong University in China. BN models can detect and express the hidden relationships among prognostic factors and are widely used in medical research fields. Furthermore, Demichelis *et al.* ^13^ proposed an extension of the well-known Naïve Bayes classifier—which accounts for biological heterogeneity in a probabilistic framework—that relies on Bayesian hierarchical models to develop a model with an accuracy of 0.65. Our model correctly classified 50 patients with survival time ≤6 months and 49 who survived >6 months, leading to a model accuracy of 81.15%. Additionally, the AUC of the ROC for the BN model was 78.1%. Therefore, we obtained a higher TPR with a given FPR, meaning that we obtained higher prediction accuracy with lower risk.

Table 2 lists the results of LR on survival time with the stepwise backward algorithm. The results showed that T stage, N stage, M stage and pathological type had a statistical significance of P < 0.1, which were used to establish ROC curve and the AUC was 87.4%. The difference of the two ROC results may be caused by the used of stepwise backward algorithm on LR, while BN analysed the whole factors.

Table 3 lists prognostic factors ranked by importance measures calculated using seven kinds of CIM: MBM, MRAW, MFV, MRRW, MAD, MMAW, and MMFV. The Birnbaum importance defines the importance of a given component as the probability that this component is critical to the functioning of the system^14^. The MBM accounts for the absolute deviation of each component state from the actual value in a multi-state system. A high value for MBM indicates that the reliability is highly-sensitive to perturbations in the state of a component. From this perspective, the value obtained for surgical type was highest, meaning that surgery type was the most significant factor dictating the prognosis of GBC patients. Meanwhile, MBM values for sex and age factors were small, meaning that they had a slight influence on patient prognosis.

The RAW measure quantifies the maximum percentage increase in system reliability generated by a particular component and it can be extended to a multi-state case. The MRAW adopts the existing condition perspective, and indicates which component is likely to improve the system performance the most, after it has been replaced by a better performing component^15^. This approach identified the M stage as the most significant factor influencing the prognosis of patients with GBC.

The FV importance measure quantifies the maximum decrement in system reliability caused by a particular component, while the RRW measures the potential damage caused to the system by a particular component. Equations (4) and (5) show that mathematical calculations can transform MFV into MRRW, affording them the same importance ranks. This approach identified the value for T stage as the largest, implicating the T stage as the most important factor underlying the prognosis of patients with GBC.

MAD, MMAW, and MMFV are alternative CIMs that account for the impact a given component has on system reliability, the perturbation of system reliability when a component state changes, and the probability that such changes occur. In other words, the MAD, MMAW, and MMFV measures account for both prior and posterior probabilities. From this perspective, the N stage had the most significant effect on the prognosis of patients with GBC. Additionally, sex and age factors had the smallest influence on GBC prognosis regardless of the importance measures selected.

The BN model depicts the dynamic and static characteristics of the dataset and expresses all the information in it. According to our model, N stage, liver infiltration, T stage, M stage, and surgical type were all dependent variables in survival time prediction. Other previous studies have considered the stage of cancer as the most significant factor for survival time^16^. GBC discovered incidentally has a better prognosis compared with patients with preoperative suspicion of GBC because of an earlier stage at incidental discovery^17^. The median survival times of GBC patients with M stages of M_0_ and M_1_ were 9.33 months and 2 months, respectively, with this difference statistically significant. The median survival times of GBC patients with T stages of T_3_ and T_4_ were 8 months and 2.67 months, respectively. The median survival times of GBC patients with N stages of N_0_, N_1_, and N_2_were 39.3 months, 4 months, and 2.67 months, respectively, with these differences statistically significant. The median survival times of GBC with R0 resection and R1/2 resection were 25.0 months and 3 months, respectively, with this difference statistically significant.

Table 3 lists prognostic factors ranked by importance measures and shows that the surgical type and TNM stage are the most significant factors among these factors, consistent with previous studies^16–18^. Table 4 lists prognostic factors analysed by Cox regression. The results showed that the surgical type, N stage, M stage and pathological grade were all independent risk factors (p < 0.05) for prognosis of GBC, which are almost same with the results of importance analysis. Maybe the lack of data for T0–2 causes the small diffidence between the two methods, but what we have confirmed is that the surgical type, NM stage are the most significant factors. And understandably, different surgical types—including radical surgery and palliative surgery—lead to different outcomes for GBC patients, with curative resection prolonging survival. The advent of GBC stage has facilitated an improvement in survival rates, with patients at different stages undergoing different therapies. Stages 0–III are potentially resectable with curative intent, while stage IV is not because of distant metastases^19^.

Briefly, we have used BN combined with importance measures to indentify the key prognostic factors influencing patient survival following surgery for GBC and compared with the Cox regression results. Our data support the use of BN as an effective tool for medical data mining and show that importance measures can be applied to analyse the influence of variables related to a target^12^. Surgical type and TNM stage are significant predictive factors of survival time for patients with GBC. However, sufficient patient data are needed to achieve a high predictive accuracy^20^. Our study employed only 13 attribute variables in the BN model, with 366 patient records in the dataset. Therefore, additional and complete clinical records of patients with GBC should be collected for future research.

## Materials and Methods

### Patients and data collection

The original medical records of 438 patients (Supplementary Table S1) who had undergone surgical procedures for the treatment of GBC were collected from the First Affiliated Hospital of Xi’an Jiaotong University in China from January 2008 to December 2012, with follow-up data was available until October 2014.

The patient dataset was established with 15 categories: jaundice, liver infiltration, pathological type, shape, pathological grade, T stage, N stage, M stage, age, surgical type, blood loss, surgical time, sex, survival state and survival time (Table 5). Patients were assessed for TNM stage according to the American Joint Committee on Cancer (7th edition)^21^.

**Table 5:** Standard description of data.

ID	Variables	General description	Values	Type
1	Jaundice	N	0	Discrete
		Y	1	
2	Liver Infiltration	N	0	Discrete
		Y	1	
3	Pathological Type	Adenocarcinoma	1	Discrete
		Non-adenocarcinoma	2	
4	Shape	Protuberance	1	Discrete
		Infiltration	2	
5	Pathological Grade	well	1	Discrete
		moderate	2	
		poor	3	
6	T Stage	Tis	0	Discrete
		T_1a_	1a	
		T_1b_	1b	
		T_2_	2	
		T_3_	3	
		T_4_	4	
7	N Stage	N_0_	0	Discrete
		N_1_	1	
		N_2_	2	
8	M Stage	M_0_	0	Discrete
		M_1_	1	
9	Age (year)		29–50, 51–70, 71–86	Continuous
10	Surgical Type	Radical Surgery (R0)	1	Discrete
		Palliative Surgery (R1/2)	2	
11	Blood Loss	≤1000 ml	0	Discrete
		>1000 ml	1	
12	Surgical Time (hour)		0.25–3, 3.01–10.25	Continuous
13	Sex	Male	1	Discrete
		Female	2	
14	Survival State	Survival	0	Discrete
		Death	1	
		Lost follow-ups	2	
15	Survival Time (month)		0.03–6, 6.1–80.47	Continuous

Jaundice was defined by the serum bilirubin level exceeding 32.4 umol/L (2 mg/dL).

Clinical end-points and measurements included imaging examination such as abdominal ultrasound, Computed Tomography (CT) and Magnetic Resonance (MR) scan, and assaying serological tumor markers, which included the determination of carbohydrate antigen 125 (CA-125), carbohydrate antigen 19-9 (CA19-9) and carcinoembryonic antigen (CEA).

### Indications for surgery

Different surgical procedures were performed based on the results of exploratory surgery and intraoperative pathological examination. In patients with advanced GBC either without involvement of the liver or with minimal liver infiltration, wedge resection of the gallbladder bed/segment IVb/V resection and regional/extended lymph node dissection was performed. When massive invasion of the liver was diagnosed, major hepatectomy procedures—such as right hemihepatectomy or right trisectionectomy—were performed. When tumours involved the extrahepatic bile duct or bulky regional lymph node metastasis near the bile duct was found, common bile duct resection was performed. Peritoneal seeding, bulky lymph node involvement, or para-aortic lymph node involvement were regarded as contraindications for surgery. HPD was considered in patients with the following conditions: (1) lower bile duct involvement, (2) pancreatic infiltration, (3) duodenal infiltration, or (4) bulky retropancreatic lymph node metastasis. Gastric resection was performed in cases of macroscopic infiltration.

Palliative surgical interventions were performed when en bloc tumour removal could not be achieved because of distant metastasis, peritoneal seeding, positive para-aortal lymph node metastasis, widespread tumour invasion, or other patient complications precluded aggressive surgery. For palliative surgery cases, biliary tract drainage was performed once jaundice or biliary tract invasion occurred.

### Follow-up

Survival time was calculated as time from surgery until patient death, when the patient was lost to follow-up, or to the end of the follow-up period for patients who remained alive when the study ended in October 2014. The follow-up interval was 6 months. Overall survival (OS) was calculated using all 438 cases within the dataset. Follow-up studies identified death in 61.9% of patients (271), while 16.4% of patients (72) were lost to follow-up. The remaining 21.7% of patients (95) remained in stable condition in October 2014.

### Bayesian network

BN is recommended as a comprehensive method of indicating relationships between variables in medical domains when conditions of causality and conditional independence are involved^22^. Formally, a BN includes nodes, edges, and conditional probability. The nodes represent random variables. Each edge represents the cause-and-effect relationship between two nodes. The conditional probability table will quantitatively express the interdependence between nodes.

Through the application of the Bayes theorem, BN is used to obtain the probabilities of unknown variables from known evidence and probabilistic relationships. Duda and Hart put forward a form of Naïve Bayes classifier (NB) based on Bayes formula in 1973. In the NB model, all attributes are conditionally independent to the class variable. Friedman *et al.* ^23^ proposed a tree augmented naïve Bayes (TAN) method which reduces the hypothesis of any attribute that is independent another in the NB classifier based on the dependent relationship of the attributes. Recently, Udelnow*et al.* ^24^ introduced the BN for cancer to predict outcome following multi-organ resection. Si *et al.* ^25^ established a breast cancer diagnosis model to identify tumour markers based on BN using a real-world database.

### Prognostic model based on Bayesian network

A total of 366 individuals whose survival state was 0 or 1, and these patients were used to establish and test the BN model. First, the survival state was excluded from 438 original dataset as survival time was the predictive variable. Next, because BN can only deal with discrete variables, continuous prognostic factors were converted into discrete values on the basis of data features and medical advice. Age was divided into three intervals of 29–50, 51–70, and 71–86 years. Surgical time was divided into two intervals of ≤3 and >3 hours based on medical suggestion. Survival time was divided into two intervals of ≤6 and >6 months according to the median survival time of 5.7 months.

To establish the BN model and test its performance, the dataset of 366 patients with GBC was stochastically divided into two cohorts using the rand function in Microsoft Excel. Two-thirds (244) of the patients formed the training dataset (Supplementary Table S2) to establish the model and the remaining 122 individuals (Supplementary Table S3) were considered as the testing dataset to test the model.

**Figure 3:**
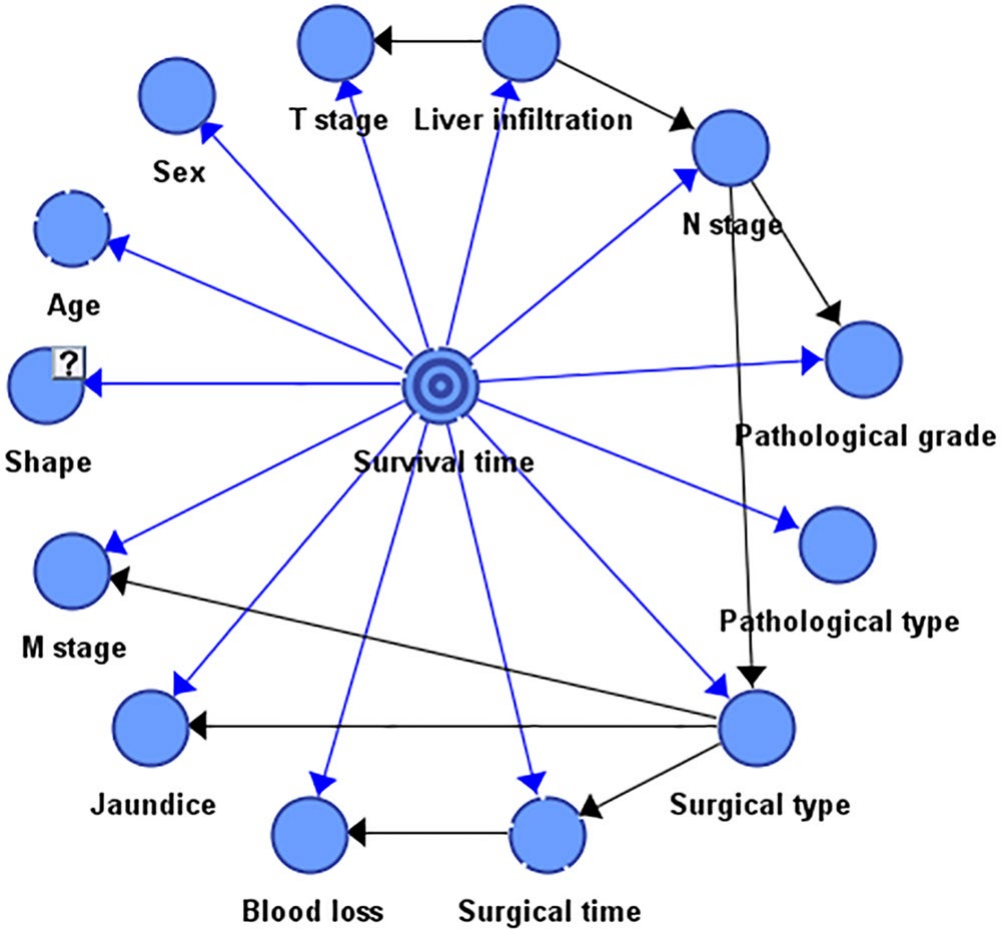
Bayesian network model for prognostic factors.

In the datasets, survival time was set as the target variable to be predicted, while other factors were considered as attribute variables that affected the state of the target variable. Then the prognostic BN model was established using the TAN algorithm implemented automatically by BayesiaLab. The TAN algorithm^23^ includes four steps: (1) Compute the mutual information function between variables, (2) Build a complete undirected graph, (3) Build a maximum weighted spanning tree, (4) Transform the resulting undirected tree to a directed one by choosing a root variable and setting the direction of all edges to be outward from it. The cause-and-effect relationships among these attribute variables are shown in Fig. 3.

### Confusion matrix and ROC curve

Confusion matrix is a tool used to evaluate the credibility of a prognostic classification model. The columns represent the actual condition, while the rows represent the predicted results of the classifier. True positive (TP) and true negative (TN) values describe correctly-classified instances. Meanwhile, false positive (FP) totals the negative instances misclassified as positive, and false negative (FN) totals the quantity of positive instances misclassified as negative.

Model reliability is defined as the values along the major diagonal of the total instances. Meanwhile, partial reliabilities are calculated by TP/(TP + FP), FP/(TP + FP), FN/(TN + FN), and TN/(TN + FN).

Model accuracy is defined by the following equation.

1 $${\rm{Accuracy}}=\frac{{\rm{TP}}+{\rm{TN}}}{{\rm{TP}}+{\rm{FP}}+{\rm{TN}}+{\rm{FN}}}$$

However, accuracy may sometimes not be the appropriate measure when the number of negative and positive cases varies widely. Considering this condition, the ROC curve and the AUC were calculated to measure the overall performance of the classification model.

The TPR of the classifier is estimated as TP/(TP + FN). The FPR of the classifier is estimated as FP/(TN + FP)^26^. ROC graphs are two-dimensional graphs in which TPR is plotted on the *Y* axis and FPR is plotted on the *X* axis. For the ROC curve, if the curve approaches the counter-diagonal line, the attribute variables have few judgment values for the target variable. Contrastingly, if the curve is far from the line, the attribute variables will have great value for the target variable.

### Importance measures

The concept of importance measures was first introduced by Birnbaum^27^ to quantify the contribution of individual components to total system performance. Nowadays, importance measures are widely used to identify the key factors within an engineering system^14, 28^. So we applied some importance measures to evaluate the influence of covariates on survival from different aspects and compared the results with the traditional Cox regression analysis.

The composite importance measures (CIM)^29^ was applied to calculate the importance of factors affecting the survival time of patients with GBC. The CIM is extended from different aspects to comprehensively evaluate the roles of different factors.

The CIM generalization for Birnbaum importance (MBM) can be expressed as

2 $${{\rm{MBM}}}_{{{\rm{V}}}_{{\rm{i}}}^{{\rm{j}}}}^{{\rm{S}}}=\frac{1}{{\omega }_{i}-1}\sum _{{\rm{j}}={\rm{1}}}^{{\omega }_{i}}|{\rm{P}}({\rm{S}}=0|{{\rm{V}}}_{{\rm{i}}}={\rm{j}})-{\rm{P}}({\rm{S}}=0)|$$

where S represents the survival time, P(S = 0) represents the prior probability of survival time, V_i_ represents the covariates with ω_i_ candidate states {1, …, j, …, ω_i_} which is also called prognostic factors, the p(S = 0|V_i_ = j) represents the posterior probability which reflect the change of survival time under the change of covariates V_i_ state. So, the influence of prognostic factors on survival time is determined by this equation.

The CIM generalization for reliability achievement worth (MRAW) was calculated as follow:

3 $${{\rm{MRAW}}}_{{\rm{i}}}=1+\frac{1}{{\omega }_{{\rm{i}}}-1}\sum _{{\rm{j}}=1}^{{\omega }_{i}}{\rm{\max }}(0,{\beta }_{{\rm{ij}}})=1+\frac{1}{{\omega }_{{\rm{i}}}-1}\sum _{{\rm{j}}=1}^{{\omega }_{{\rm{i}}}}{\rm{\max }}(0,\frac{{\rm{P}}(S=0|{{\rm{V}}}_{{\rm{i}}}={\rm{j}})-{\rm{P}}({\rm{S}}=0)}{{\rm{P}}({\rm{S}}=0)})$$

The CIM generalization for Fussell-Vesely importance (MFV) was expressed as follows:

4 $${{\rm{MFV}}}_{{\rm{i}}}=\frac{1}{{\omega }_{{\rm{i}}}-1}\sum _{{\rm{j}}=1}^{{\omega }_{{\rm{i}}}}{\rm{\max }}(0,-{\beta }_{{\rm{ij}}})=\frac{1}{{\omega }_{i}-1}\sum _{{\rm{j}}=1}^{{\omega }_{{\rm{i}}}}{\rm{\max }}(0,\frac{{\rm{P}}({\rm{S}}=0)-{\rm{P}}({S=0|V}_{{\rm{i}}}={\rm{j}})}{{\rm{P}}({\rm{S}}=0)})$$

The CIM generalization for reliability reduction worth (MRRW) was calculated as follow:

5 $${{\rm{MRRW}}}_{{\rm{i}}}=\frac{1}{1-{{\rm{MFV}}}_{{\rm{i}}}}$$

The CIM generalization for mean absolute deviation (MAD) was calculated as follow:

6 $${{\rm{MAD}}}_{{\rm{i}}}=\sum _{{\rm{j}}}{{\rm{P}}}_{{\rm{ij}}}|{\rm{P}}({\rm{S}}=0|{{\rm{V}}}_{{\rm{i}}}={\rm{j}})-{\rm{P}}({\rm{S}}=0)|$$

The CIM generalization for mean multi-state reliability achievement worth (MMAW) was calculated as follow:

7 $${{\rm{MMAW}}}_{{\rm{i}}}=1+\sum _{{\rm{j}}}{{\rm{P}}}_{{\rm{ij}}}\,{\rm{\max }}(0,{\beta }_{{\rm{ij}}})$$

The CIM generalization for mean multi-state Fussell-Vesely (MMFV) was calculated as follow:

8 $${{\rm{MMFV}}}_{{\rm{i}}}=\sum _{{\rm{j}}}{{\rm{P}}}_{{\rm{ij}}}\,{\rm{\max }}(0,-{\beta }_{{\rm{ij}}})$$

### Statistical analysis

SPSS 13.0 for Windows (SPSS Inc., Chicago, IL, USA) was used for statistical analyses. BayesiaLab (Bayesian Limited Company, France) was used to establish a BN. Microsoft Excel was used to prepare the training and testing datasets. All continuous variables were transformed into discrete variables. Survival rates were calculated according to the Kaplan-Meier method and differences were measured with the Log-rank test. Moreover, prognostic multivariate analysis was analyzed by Cox regression and importance measures. Statistical significance was set at P < 0.05.

### Ethics statement

This study was approved by the Ethics Committee of the First Affiliated Hospital of Xi’an Jiaotong University. All patients gave written informed consent to participate. The ethics committee approved this consent procedure. Data did not contain any information that could identify the patients. All methods were performed in accordance with the relevant guidelines and regulations.

## Electronic supplementary material

Supplementary Tables.
