# Supplementary material for: Analysis of prognostic factors for survival after surgery for gallbladder cancer based on a Bayesian network
Source: Sci Rep. 2017 Mar 22;7:293. doi: 10.1038/s41598-017-00491-3 (PMC5428511; doi:10.1038/s41598-017-00491-3)
Supplement: Supplementary file 1 — Supplementary Tables. [file 41598_2017_491_MOESM1_ESM.pdf]

Title page

Title：Analysis of prognostic factors for survival after surgery for gallbladder cancer based on a Bayesian network

Authors: Zhi-qiang Cai[1] PhD, Peng Guo[1] ME, Shu-bin Si[1,*] PhD, Zhi-min Geng[2,+] MD, Chen Chen[2] MD, Long-long Cong[2] MD

Address:

1 Department of Industrial Engineering, School of Mechanical Engineering, Northwestern Polytechnical University, Xi’an 710072, Shaanxi, China.

2 Department of Hepatobiliary Surgery, First Affiliated Hospital, Xi’an Jiaotong University, Xi’an 710061, Shaanxi, China.

Corresponding author: Shu-bin Si[*].

Address: Department of Industrial Engineering, School of Mechanical Engineering, Northwestern Polytechnical University, Xi’an 710072, China.

Tel.: +86 (0) 139 9136 3388

Fax: +86 (0) 29 8846 0490.

E-mail: sisb@nwpu.edu.cn

Co-corresponding author: Zhimin- Geng[+].

Address: Department of Hepatobiliary Surgery, First Affiliated Hospital, Xi’an Jiaotong University, Xi’an 710061, Shaanxi, China.

Tel.: +86 (0) 137 7217 5199

Fax: +86 (0) 29 8266 8388

E-mail: gengzhimin@mail.xjtu.edu.cn

Supplementary table legends

Supplementary Table S1. Records of 438 patients with GBC after surgery.

Supplementary Table S2. Records of 244 patients with GBC after surgery for modeling.

Supplementary Table S3. Records of 122 patients with GBC after surgery for testing.

Supplementary Table S1. Records of 438 patients with GBC after surgery.

Jaundice	Liver infiltration	Gender	Surgical type	T	N	M	Pathological Grade	Pathological Type	Blood Loss	Age	Shape	Surgical time	Survival state	Survival time
1	1	1	2	4	2	1	2	1	0	65		1.5	1	1.0000
1	0	1	2	3	2	1	2	1	0	41		1.75	1	3.0000
0	1	2	2	4	2	1	3	1	0	51		1	1	2.0000
0	1	2	2	4	2	1	3	1	0	67		1	1	1.0000
0	1	2	2	4	2	1	3	1	0	56		1	1	1.5000
0	0	2	2	3	0	0	2	1	0	79	1	1	0	62.5000
0	0	2	1	3	0	0	1	1	0	64	2	2.25	0	57.8000
0	1	2	2	3	2	1	3	1	0	54		1.5	1	1.0000
1	0	2	2	3	2	1	2	1	0	66	1	1.25	1	2.0000
0	1	2	2	3	2	1	3	1	0	69		1.5	1	1.0000
0	1	2	2	4	2	0	2	2	0	62		3.75	1	1.6667
0	1	2	2	4	2	1	3	1	0	50		2	1	1.3333
1	0	2	2	3	0	0	1	1	0	78	1	2.25	0	63.5000
1	0	2	2	3	0	0	2	1	0	76	2	2	0	57.1333
0	0	1	2	3	0	0	2	1	0	74	2	1.5	1	13.0000
1	1	1	2	4	2	1	2	1	0	66		4.5	1	3.0000
0	0	2	2	3	0	0	3	1	0	56	2	3	0	79.1000
0	0	1	2	3	1	1	2	1	0	72	1	2.75	2	
0	0	1	2	3	0	0	2	1	0	59	2	3.25	0	80.4667
0	0	2	1	3	1	0	3	1	0	67	2	1.75	1	8.0000
0	1	1	2	4	1	0	2	1	0	51	1	3	1	6.6667
1	0	2	2	3	0	0	1	1	0	68		2.5	0	52.3667
0	0	2	1	0	0	0		1	0	43	2	2.25	0	54.1000
0	0	2	1	3	0	0	3	1	0	73	2	2.25	0	28.6667
1	1	2	2	4	1	0	2	1	0	48	1	3	2	
0	1	2	2	3	2	1	3	1	0	71		2.25	1	2.6667
1	0	2	2	3	0	0	1	2	0	82	2	2	1	5.0000
1	1	2	2	4	0	0	1	2	0	49	2	2.25	1	9.0000
0	0	1	2	3	1	0	2	1	0	71	1	2	1	5.0000
0	0	2	2	3	2	0	2	1	0	76	1	1.75	1	7.0000
0	1	2	2	3	0	0	2	1	0	71	1	2	1	7.3333
0	1	2	2	3	2	1	3	1	0	59		1.5	1	2.3333
0	1	2	2	4	2	1	3	1	0	60		1.5	1	1.3333
0	1	2	2	4	2	1	3	1	0	61		1.25	1	0.5000
0	1	2	2	4	2	0	3	1	0	70		1.5	1	2.3333
0	0	2	2	1a	0	0	1	1	0	81		3	0	30.1667
1	1	2	2	4	1	0	3	1	0	79		2.25	1	3.0000
0	0	1	2	3	1	1	2	1	0	48	2	4	1	5.0000
0	0	2	2	3	2	1	3	1	0	55		4	1	2.3333
0	1	2	2	3	1	1	3	1	0	60		0.5	1	0.2333
0	1	2	2	4	1	1	2	1	0	67		0.5	1	1.6667
1	1	2	2	4	1	0	3	1	0	72		4	1	2.0000
0	0	2	1	3	0	0	2	1	0	69	2	1.75	0	42.7000
0	1	2	2	4	1	1	2	2	0	61		2.75	1	2.0000
0	0	2	2	3	2	1	3	1	0	68	1	2.5	1	1.3333

0	0	1	2	3	1	0	3	1	0	67		2.5	1	3.0000
1	1	2	2	4	1	0		1	0	68		2.25	1	3.0000
0	1	1	2	4	1	1	2	1	0	61	1	1.5	1	2.0000
0	1	2	2	4	1	0	3	1	0	76	1	2.75	1	2.0000
1	0	2	2	3	2	1	2	1	0	81		3	1	4.0000
1	1	1	2	4	1	0	2	1	0	65		2.5	2	
1	0	2	2	3	1	1	3	1	0	54	1	2.75	2	
0	1	1	2	4	1	1	3	2	0	78		2	1	1.0000
0	0	2	1	3	0	0	1	1	0	61	2	3	1	3.6667
0	0	1	1	0	0	0	1	1	0	65		2	0	42.8000
1	0	2	1	0	0	0	1	1	0	78		2.25	0	26.8667
0	0	2	1	3	1	0	2	1	0	52	1	3.25	2	
0	0	1	2	3	1	1	3	1	0	64	2	2	2	
0	1	2	2	3	2	1	2	1	0	57		0.5	1	2.3333
0	1	1	2	4	2	0	2	1	0	84	1	2.5	1	1.1667
0	0	2	1	1a	0	0	1	1	0	65		2.75	0	25.4000
0	0	2	2	2	0	0	2	1	0	75	1	2.25	1	3.0000
0	0	2	2	3	1	0	1	1	0	84	1	3.25	1	15.0000
0	0	1	2	3	1	1	3	1	0	55		2.5	1	3.0000
0	0	2	2	3	1	1	3	1	0	76	1	2.25	1	1.0000
0	0	2	2	3	1	1	3	1	0	65		1.5	1	2.0000
0	0	2	2	3	2	0	2	1	0	50		1.25	2	
0	1	2	1	3	0	1	2	1	0	74	1	2.5	1	8.0000
0	1	1	2	4	2	0	3	2	0	51	1	2	1	1.6667
0	1	2	2	4	1	0	2	2	0	71	1	3.75	1	2.0000
1	0	1	2	3	2	0	2	2	0	78		3	1	10.0000
0	0	2	1	3	0	0	2	1	0	46	1	2	0	57.5000
0	0	2	1	3	0	0	1	1	0	63		2.5	0	37.7667
1	1	2	2	4	1	1	2	1	0	62		2.75	1	2.0000
0	0	2	2	1b	0	0	2	1	0	63		2.75	0	74.0667
0	0	2	2	1b	0	0	1	1	0	60	2	3	1	63.3333
0	1	2	2	4	1	0	3	1	0	42		2	1	6.0000
1	1	2	2	4	1	0	3	1	0	48	1	2	1	6.3333
0	0	2	2	3	2	0	3	1	0	60	2	2.25	1	4.3333
0	1	2	2	3	1	1	3	1	0	86	2	2.75	2	
0	1	2	2	4	1	1	2	1	0	72	1	4	1	3.0000
0	1	2	2	4	1	1	2	1	0	46	2	2.25	2	
1	1	1	2	4	2	1	3	1	0	58		0.25	1	1.0000
1	1	1	2	4	1	1	3	1	0	65		2.5	2	
1	1	2	2	4	2	0	3	1	0	49		1.25	2	
1	0	2	2	3	2	0	3	2	0	43	1	2.25	1	10.0000
0	1	2	1	3	2	0	3	2	0	65	1	2.5	1	5.6667
0	1	1	2	3	2	0	2	2	0	65	1	1	1	7.0000
0	0	2	1	3	1	0	3	1	0	39		3	1	4.0000
1	0	2	2	3	2	1	3	1	0	45	1	3.5	2	
0	0	1	2	3	2	0	2	1	0	65	1	4	1	2.0000
0	0	1	2	3	1	1	3	1	0	52	1	2.75	1	6.6667
0	0	2	1	3	2	0	3	1	0	62		2	1	6.0000

0	0	1	1	3	0	0	2	1	0	65	1	2.5	0	27.5333
1	0	2	1	3	1	0	2	1	0	62	1	4.5	2	
0	0	2	1	3	1	0	3	1	0	58	2	2	2	
1	1	2	2	4	1	0	3	1	0	80	1	2.25	2	
0	0	2	2	3	2	0	2	1	0	53	1	2	1	4.3333
0	1	2	2	4	1	1	3	1	0	50		1.25	1	1.3333
0	1	2	1	3	2	0	3	1	0	73	1	3.75	2	
0	0	2	2	3	1	0	3	2	0	57	1	3	2	
0	0	2	2	3	0	0	1	1	0	57		1.25	0	69.8667
0	0	2	2	3	0	1	2	1	0	71	1	3	2	
0	1	2	2	4	2	1	3	1	0	56	1	2	1	1.0000
0	1	1	2	3	0	0	2	1	0	51	1	5.75	1	23.3333
0	1	2	2	3	0	0	2	1	0	54	1	2.25	1	20.0000
0	0	2	1	3	1	0	2	1	0	65	1	2.25	2	
0	1	1	2	4	1	0	2	1	0	58		2.75	1	5.3333
1	1	2	2	4	1	0	3	1	0	46		2.75	0	29.1333
0	1	2	2	3	1	1	2	1	0	57		2.25	2	
1	0	2	2	3	2	0	3	1	0	65	1	2.5	1	2.0000
0	1	2	2	3	1	1	3	1	0	55	1	3.25	1	1.6667
0	1	1	2	4	2	1	3	1	0	59		3	1	1.3333
1	1	1	2	4	1	1	3	1	0	49	1	2	1	0.0333
0	1	1	2	4	2	0	1	1	0	68		4.75	1	5.0000
0	1	2	2	4	1	0	3	2	0	74		2.5	1	5.0000
0	1	2	2	4	2	0	3	2	0	61	2	3.25	1	1.6667
1	1	2	2	4	1	1	2	2	0	71	1	2.75	1	1.6667
0	0	2	2	3	1	0	3	1	0	49	1	2.25	2	
0	1	1	2	3	2	0	3	1	0	50		3	1	8.0000
0	1	2	2	3	1	1	1	1	0	61	1	2.25	1	0.3000
0	1	2	2	4	1	1	2	1	0	46		3	1	4.0000
0	0	2	2	3	2	1	2	2	0	76		1	1	3.0000
0	0	2	1	1b	0	0	2	1	0	61	1	2.75	0	59.7333
0	0	1	1	3	0	0	2	1	0	72	1	3.5	1	20.0000
0	0	2	1	3	0	0	2	1	0	44	2	4	0	56.6667
1	1	2	2	4	1	0	2	1	0	58		3	1	12.1667
0	1	1	2	4	2	0	2	1	0	66		2	1	2.3333
0	0	2	1	1b	2	0	3	2	0	52	2	2.75	0	34.2333
0	1	2	2	3	1	1	3	2	0	65	2	3.75	1	2.0000
0	1	2	2	4	2	1	2	2	0	65	1	1	1	3.0667
0	1	2	2	3	2	1	3	2	0	63	1	2.5	1	3.5000
1	0	2	1	3	0	0	3	2	0	64	2	3.75	1	4.8333
0	0	2	1	3	1	0	2	1	0	63		3	1	7.0000
0	1	1	1	4	1	0	2	1	0	37	1	5.25	1	32.0000
0	0	1	1	3	0	0	2	1	0	78	1	3.25	0	32.3000
1	0	2	1	3	1	0	3	1	0	56		2.5	1	1.3333
1	1	2	2	4	1	0	2	1	0	51		3	1	8.0000
1	1	2	2	4	1	0	3	1	0	48	1	2.75	2	
1	1	2	2	4	1	0	3	1	0	53	1	5	1	9.0000
1	1	1	2	3	1	1	2	1	0	39	2	2	1	5.0000

1	1	2	2	3	1	1	2	1	0	46		3.75	1	5.5000
1	0	2	2	3	1	1	3	1	0	72	2	2.75	1	1.3333
0	0	1	2	3	2	1	3	1	0	62	1	1.75	1	1.0000
1	1	2	2	4	2	0	2	1	0	49	2	2.75	1	6.0000
1	1	2	2	4	2	0	2	1	0	70	1	3	0	30.0667
1	1	2	2	4	2	1	2	1	0	68		1.75	1	6.1667
0	1	1	2	4	2	1	2	1	0	55	1	2.75	1	2.6667
0	1	2	2	4	2	0	3	1	0	69		2	2	
1	1	2	2	4	1	0	3	2	0	61	1	2.75	1	3.3333
0	0	2	1	3	0	0	2	1	0	48		3.25	0	30.1333
0	1	2	2	3	2	0	2	1	0	50	1	4	1	6.0000
0	0	2	1	3	1	0	3	1	0	70	1	1.5	1	6.9000
0	1	2	2	4	1	0	3	1	0	70		2	1	2.6667
1	1	2	1	4	1	0	2	1	0	58		2	1	7.5333
0	0	2	2	3	1	1	1	1	0	73		1.75	1	10.0000
0	1	1	2	4	2	1	3	2	0	50		2.5	1	1.5000
0	0	2	2	3	1	0	3	1	0	71	1	0.5	1	9.0000
0	0	2	2	3	1	1	1	1	0	55		6	1	7.0000
0	0	2	1	3	0	0	2	1	0	68	2	2.75	0	50.3333
0	0	2	1	3	0	0	2	1	0	71	2	3	0	45.5667
0	0	1	1	3	0	0	2	1	0	67	2	2.25	0	29.9667
0	1	2	1	3	1	1	2	1	0	72	1	3.25	2	
0	1	2	2	4	1	0	3	1	0	58		2.5	1	14.0000
1	1	2	2	4	1	0	2	1	0	65		4	2	
0	1	2	2	4	1	0	3	1	0	65		3.25	1	2.0000
1	1	2	2	4	1	0	3	1	0	51		2.75	1	2.0000
0	1	2	2	4	1	0	2	1	0	86	1	2.25	1	4.0000
0	1	1	2	3	1	1	3	1	0	61		1.25	1	1.2000
1	1	1	2	3	1	1	3	1	0	69		3.25	1	12.0000
1	1	2	2	3	2	1	3	1	0	60		1.75	1	1.0000
0	1	2	2	3	1	1	2	1	0	52		1.75	1	3.0000
0	0	2	2	3	2	0	3	1	0	53	2	3.5	1	2.0000
0	0	1	2	3	2	1	3	1	0	50	2	2.25	2	
1	1	2	2	3	2	1	3	1	0	44		3	1	1.6667
0	0	2	2	3	2	1	1	1	0	61	1	3.75	1	3.0000
0	1	2	2	4	1	1	3	1	0	41		3.25	1	3.0000
0	1	1	2	4	2	0	2	1	0	63	1	3.5	1	3.0000
0	1	2	2	4	2	1	3	1	0	57	1	3	0	51.4667
0	1	2	2	4	2	0	3	1	0	69	1	3.75	2	
0	1	1	2	4	1	1	2	1	0	59	1	2.75	1	3.0000
0	1	2	2	4	2	1	3	1	0	57		2.75	1	1.3333
1	1	2	2	4	2	0	3	1	0	77	1	3	1	0.9000
0	1	1	2	4	1	1	3	1	0	71		2.25	1	1.0000
0	1	2	1	3	2	0	2	1	0	63	1	6	1	9.3333
0	1	2	2	4	1	0	2	2	0	83		1.75	2	
0	1	2	2	3	2	1	2	2	0	45	2	2.75	1	4.0000
0	0	2	2	3	0	0	2	1	0	68	1	1.75	0	52.9333
0	0	2	1	3	1	0	3	1	0	51		2.5	0	67.4000

0	0	2	1	3	1	0	1	1	0	75		2.75	0	48.0000
0	1	2	2	4	1	0	3	1	0	71		2	2	
1	1	2	2	4	1	0	2	1	0	60		2	1	8.0000
1	1	1	2	4	1	0	3	1	0	64	1	4.75	1	1.0000
0	1	2	2	4	1	0	2	1	0	42		4.5	2	
1	1	1	2	4	1	0	3	1	0	37	1	3	2	
1	0	2	2	3	2	0	3	1	0	63	1	3	2	
0	0	2	2	3	2	0	2	1	0	69	1	2	1	12.1667
0	0	1	2	3	2	1	3	1	0	64		2.75	1	0.6667
0	1	2	2	3	2	1	2	1	0	45	1	3.5	2	
0	1	1	2	3	1	1	2	1	0	58	1	3.75	1	7.2333
0	0	1	2	3	2	1	3	1	0	67	1	2.75	1	1.0000
1	1	2	2	4	2	0	3	1	0	63		2.5	1	2.0000
1	1	2	2	4	2	0	3	1	0	49	1	3	1	2.6667
0	1	2	1	3	2	0	3	1	0	78	1	4.25	2	
0	0	2	2	3	1	0	2	1	0	64	1	2.5	1	5.6667
0	0	2	1	3	0	0	2	1	0	55		3.5	0	36.8333
0	0	1	1	3	1	0	3	1	0	66	1	4	2	
0	0	1	2	3	1	1	3	1	0	83	2	2.25	1	2.5000
0	0	2	1	3	0	0	1	1	0	64	1	3	0	47.8000
0	1	2	2	3	2	0	3	1	0	69	1	2	2	
0	1	2	2	4	1	1	2	1	0	60		1.75	1	2.0000
0	1	1	2	3	2	1	3	1	0	65		1.25	2	
0	1	1	2	3	1	1		2	0	75		3	1	2.0000
0	1	2	2	4	2	0	3	2	0	38		2.5	1	4.3333
0	0	1	2	3	0	0	2	1	0	51		1.75	0	77.1000
0	0	2	2	3	0	0	3	1	0	71		1.25	0	74.9333
1	0	2	2	3	2	0	2	1	0	58		1.5	1	5.0000
0	1	2	2	3	2	0	2	1	0	54		1.75	2	
0	0	2	2	3	2	0	2	2	0	59	1	2.5	1	5.6667
0	1	1	2	4	2	0	3	2	0	68	2	3	1	3.0000
0	0	2	1	3	0	0	1	1	0	56	1	4.25	0	51.4333
0	0	2	2	3	1	0	3	1	0	60	1	2.25	1	14.0000
1	1	2	2	4	1	0	2	1	0	83		4.25	1	0.6667
0	1	2	2	4	1	0	3	1	0	49	1	3.5	2	
1	1	2	2	4	1	0	2	1	0	75	2	2	1	2.6667
0	1	2	2	3	2	1	3	1	0	59	2	5.75	1	1.0000
0	0	1	2	3	2	0	2	1	0	75	1	2.75	1	2.5000
1	0	2	2	3	2	0	3	1	0	54	1	2.5	2	
0	1	1	2	4	1	1	3	1	0	64		3.5	1	1.3333
0	1	1	2	4	2	0	3	1	0	72		2.75	1	3.0000
0	1	2	2	4	2	1	2	1	0	65		3	1	1.1667
0	1	2	1	3	2	0	2	1	0	60	1	5	1	1.0000
0	0	2	1	3	0	0	3	1	0	49		4.25	0	23.4333
0	0	2	1	3	1	0	3	1	0	54	1	4.5	1	4.0000
1	1	1	2	4	2	0	2	1	0	54	1	2.5	1	5.2000
0	0	2	1	3	1	0	2	2	0	51	2	3.75	2	
0	1	2	2	4	2	0	2	2	0	78	1	4.5	1	3.0000

0	0	2	1	2	0	0	2	1	0	66		3.5	0	69.6667
0	0	2	2	3	0	0	2	1	0	82	1	1.25	0	37.2333
0	0	2	1	3	0	0	3	1	0	59		4.5	0	64.1333
0	0	2	1	3	0	0	1	1	0	50		2.5	1	22.0000
0	0	2	1	1b	0	0	2	1	0	75	1	2.5	0	51.0333
0	0	2	2	3	0	0	2	1	0	67	2	3.25	0	64.6667
0	0	2	2	3	0	0	2	1	0	69		1.75	0	23.3000
0	0	1	1	3	1	0	3	1	0	39	2	3.5	1	2.0000
1	1	1	2	4	1	0	3	1	0	63	1	3.5	1	9.0000
1	1	1	2	4	1	0	3	1	0	68	1	5	1	5.0000
0	1	1	2	4	1	0	3	1	0	66	1	1.25	1	3.6667
1	0	2	2	3	2	0	2	1	0	45		2.25	1	1.0000
0	0	2	2	3	2	1	3	1	0	37	2	4.25	2	
0	0	2	2	3	2	0	2	1	0	68	2	1.5	1	10.0000
0	0	2	2	3	2	0	3	1	0	70	1	2.25	2	
0	1	1	2	4	1	1	2	1	0	71	1	4.5	1	2.3333
1	1	1	2	4	1	1	2	1	0	75	1	3	2	
0	1	2	2	3	1	1	3	2	0	57		3	2	
0	1	2	2	4	2	1		2	0	75		1.25	1	0.4333
0	0	2	1	2	0	0	3	2	0	49		3	1	23.3333
0	1	1	2	4	1	0	2	2	0	65		4	1	3.3333
0	0	2	1	3	0	0	1	1	0	62		4.75	1	44.3333
0	0	2	1	3	0	0	2	1	0	59		4	0	38.3333
0	0	1	1	3	0	0	3	1	0	41	1	5.25	0	28.5000
0	1	2	2	4	0	0	3	1	0	65	1	5	1	38.0000
1	1	2	2	4	2	0	1	1	0	56	2	2.25	2	
0	0	2	1	3	0	0	3	1	0	71	2	3	1	7.0000
1	0	2	1	3	1	0	3	1	0	78	1	5	1	7.0000
0	1	1	2	4	1	0	3	1	0	66	1	2.25	1	10.0000
1	1	2	2	4	1	0	1	1	0	56	1	2.75	2	
1	0	2	2	3	2	1	3	1	0	65	1	3	1	3.0000
0	0	2	2	3	2	0	3	1	0	73	1	3.25	1	7.0000
1	1	2	2	4	2	0	3	1	0	66		4	1	1.0000
0	1	1	2	4	1	1	2	1	0	62	2	3	1	2.6667
0	1	1	2	4	1	1	3	1	0	56	1	4.5	1	6.0000
1	0	1	1	3	0	0		2	0	61		1.75	0	75.2000
1	0	1	1	3	0	0	1	1	0	60	2	1.25	0	65.7000
0	0	2	1	3	0	0	1	1	0	75	1	3.5	0	77.8000
0	1	2	2	4	1	0	2	2	0	47	1	4	1	7.0000
1	1	1	2	4	1	0	2	2	0	71	1	2.75	2	
0	1	2	2	3	2	1	3	2	0	59	2	2.5	1	1.0000
0	0	2	1	3	1	0	2	1	0	61		4.5	0	22.0667
0	1	2	1	3	0	0	3	1	0	39		3.5	0	66.4667
1	0	1	1	3	0	0	2	1	0	55	2	3	0	63.5000
0	0	1	1	3	0	0	2	1	0	65	1	2.5	0	51.0000
0	0	2	1	3	0	0	3	1	0	66	1	4	0	41.3000
0	1	2	2	4	1	0	3	1	0	87		2.25	2	
1	1	2	2	4	1	0	2	1	0	55	1	4	1	4.0000

1	1	2	2	4	1	0	3	1	0	66		2.25	1	2.0000
0	1	2	2	4	1	0	3	1	0	60		2.5	1	1.0000
1	1	2	2	3	2	1	2	1	0	71		2.75	2	
1	0	2	2	3	2	1	2	1	0	61	2	3	1	0.6667
0	0	2	2	3	2	0	2	1	0	54	1	3	2	
0	1	1	2	4	2	0	3	1	0	66		2	1	2.0000
1	1	2	1	3	2	0	2	1	0	60	1	5.25	1	9.0000
0	0	2	1	3	0	0	3	2	0	46		2.75	0	23.2000
0	0	2	1	3	0	0	1	1	0	65	1	1.5	0	55.2333
0	0	2	1	1b	0	0	1	1	0	76		2.5	0	24.6333
0	1	1	1	3	0	0	2	1	0	68	2	3.5	0	40.7333
0	1	2	2	4	1	0	1	1	0	73		2.75	1	3.0000
0	1	2	1	4	1	0	2	2	0	65		5.25	1	12.1667
1	1	1	2	4	1	1	2	2	0	59		6	2	
0	0	2	1	1a	0	0	3	1	0	52	1	6.25	0	39.1667
0	0	2	1	3	0	0	2	1	0	62		2.75	1	42.6000
0	0	2	1	3	1	0	3	1	0	71	1	4.75	1	7.0000
0	0	2	1	3	1	0	3	1	0	52	1	4	0	33.6333
0	1	2	1	4	2	0	2	1	0	72		2.25	1	3.0000
0	0	2	1	3	0	0	2	1	0	29	2	1.75	0	54.7333
0	0	2	1	3	0	0	2	1	0	58	1	4	0	47.6333
1	0	1	2	3	1	0	2	1	0	83		4.75	1	5.4667
0	0	1	1	3	1	0	3	1	0	62	2	2.25	0	66.7000
1	0	2	1	3	1	0	3	1	0	74	1	5.5	1	12.5000
1	1	1	2	4	1	0	2	1	0	51		3.5	1	1.0000
1	1	2	2	4	1	0	2	1	0	68	1	5.5	1	2.0000
1	1	2	2	4	1	0	3	1	0	67	1	3.25	1	2.0000
0	0	2	2	3	2	0	2	1	0	56		2.5	1	3.6667
0	1	2	2	3	2	0	2	1	0	48	1	4	1	6.0000
0	1	2	2	3	1	1	3	1	0	60		3	1	18.8333
0	1	2	2	3	2	1	3	1	0	64	1	2.5	1	3.0000
0	1	2	2	4	1	1	3	1	0	62	1	4.75	1	1.6667
0	1	1	1	3	2	0	2	1	0	64		7	1	5.0000
0	1	2	1	4	2	0	2	2	0	61	2	6.5	0	21.8667
0	0	2	1	3	1	0	3	2	0	40		4	2	
0	1	2	2	3	2	0	3	2	0	62	1	3.25	1	10.0000
0	1	2	2	4	1	1	3	2	0	51	1	4.25	1	3.0000
0	0	2	1	3	2	0	3	1	0	63	1	4.75	1	5.6667
0	0	2	1	3	0	0	3	1	0	42	2	5.75	0	51.4667
0	0	2	1	3	0	0	2	1	0	59	2	5.25	0	48.6333
0	0	2	1	3	0	0	2	1	0	62	1	4	1	11.0000
0	0	2	1	3	1	0	2	1	0	62	2	3.75	1	16.0000
0	0	2	1	3	1	0	2	1	0	44	2	4	2	
0	0	2	1	3	1	0	2	1	0	46	2	3.75	2	
0	1	2	1	4	0	0	3	1	0	45	1	6.75	1	3.3000
0	1	2	2	4	2	1	3	1	0	75		5	1	1.0000
0	0	2	1	3	2	0	3	1	0	53	2	3.5	1	12.1667
0	1	2	2	4	2	0	3	2	0	58	2	2.5	1	3.0000

1	1	2	2	4	1	0	3	2	0	66	2	2	1	3.0000
1	0	2	1	2	0	0	1	1	0	54		4	0	38.1333
0	1	2	1	3	0	0	1	2	0	61	2	2.5	0	55.4667
0	0	1	1	3	0	0	2	1	0	37		3.5	0	28.9000
1	0	2	1	1a	0	0	1	1	0	83	2	4.75	1	31.3333
0	1	1	1	3	0	0	3	1	0	56	1	4.5	0	30.2000
1	1	2	2	4	1	1	2	1	0	81		3	1	2.3333
0	0	1	1	3	1	0	2	1	0	73	2	4	2	
1	1	1	1	3	1	0	2	1	0	54	1	3.5	0	39.7667
0	1	1	2	4	1	0	2	1	0	68	1	2.75	1	4.6667
0	1	2	2	4	2	1	3	1	0	70	1	4.75	1	1.0000
0	0	2	1	3	1	0		2	0	52	2	2.5	0	31.8000
0	0	2	1	3	0	0	2	1	0	65	2	1.75	0	78.4000
0	0	2	1	3	0	0	2	1	0	77	2	4.75	0	45.7667
0	0	2	1	3	1	0	3	1	0	44	2	5.5	1	24.0000
1	1	2	2	4	2	0	3	1	0	48	2	3.75	1	2.0000
0	1	2	1	3	2	0	3	1	0	60		2.75	1	0.6667
0	1	1	1	3	0	0	3	2	0	68	1	4.5	1	2.0000
0	1	2	1	3	0	0	3	2	0	58	1	3	0	26.5000
1	1	2	2	4	1	0	3	2	0	86	2	2.25	1	2.0000
0	1	2	2	4	1	0	2	1	0	49	1	4.5	1	14.0000
0	1	1	1	3	2	0	2	1	0	66	1	2.75	1	5.0000
1	0	2	1	3	0	0	3	1	0	64	1	4.25	0	42.1333
0	0	1	1	3	0	0	2	1	0	69		3.5	0	33.3000
0	1	1	1	3	1	0	2	1	0	61	1	3.25	1	5.3333
0	1	2	2	4	1	0	2	1	0	44	1	3	2	
0	0	2	1	3	2	0	2	1	0	63	1	2.75	1	1.0000
0	0	1	1	3	1	0	2	1	0	47		4	2	
0	0	2	1	3	2	0	3	1	0	51		7	2	
0	0	1	1	3	1	0	3	1	0	75	2	4.5	0	44.9000
0	1	1	1	3	1	0	3	1	0	64		5	1	7.0000
0	0	2	1	3	1	0	2	1	0	64	1	3.25	1	6.0000
0	0	2	1	3	2	0	3	1	0	49	2	3.25	1	2.0000
0	1	2	1	3	1	1	2	1	0	66		2.5	1	1.3333
0	1	1	1	3	0	0	3	1	0	66	2	5.25	1	4.0000
1	0	1	1	3	0	0	3	1	0	71		2.75	0	23.1667
0	1	2	1	3	1	0	2	2	0	55	1	5.25	1	10.0000
0	1	1	1	3	1	0	3	2	0	73	1	3.5	1	3.0000
0	0	2	1	3	2	0	3	2	0	68		5.25	2	
1	1	1	2	3	2	0	3	2	0	78	1	4.25	1	0.6000
0	0	2	1	3	2	0	3	1	0	56		4.5	1	5.3333
0	0	2	1	1b	0	0	2	1	0	46		3	0	32.3000
0	0	2	1	3	0	0	2	1	0	61	1	4	0	54.6333
1	0	2	2	3	2	0	3	1	0	66	1	1.25	1	1.6667
1	1	1	2	4	2	1	3	1	0	57		3	1	1.0000
1	1	2	2	4	2	0	2	1	0	69	1	3	2	
0	1	2	1	3	2	0	2	1	0	57	1	5.75	1	7.6667
0	1	2	2	4	1	1	3	1	0	70	1	4.5	1	1.0000

1	1	2	1	3	1	0	2	1	0	65		6	0	39.6000
0	0	1	1	3	0	0	2	1	0	74	2	2.25	0	63.4333
0	1	1	2	4	2	1	3	1	0	79	1	5.25	1	1.3333
0	1	2	2	3	2	0	2	1	0	63	1	4	1	3.0000
0	1	2	2	4	2	0	3	1	0	36	1	3.5	1	1.6667
1	1	1	1	3	0	0	2	1	0	64	1	6.75	1	15.0000
0	0	2	1	3	0	0	2	1	0	55	1	5.75	0	43.2333
0	1	2	2	3	2	1	3	1	0	71		0.75	1	0.5000
0	0	2	2	3	2	0	2	1	0	69	1	2.25	1	6.0000
0	1	2	1	3	2	0	2	1	0	65		5	2	
0	0	2	1	3	2	0	2	1	0	64	2	3.5	2	
0	0	2	1	3	0	0	3	1	0	52		3.25	0	41.5333
0	0	1	1	3	1	0	1	1	0	62	1	6	0	29.8667
1	1	2	2	3	2	0	3	1	0	42	1	6	2	
0	1	1	1	3	2	0	3	1	0	38	1	5.5	1	11.6667
0	1	2	2	3	2	0	3	1	0	51	1	3.5	1	4.3333
1	0	2	1	3	1	0	3	2	0	51	1	5.25	1	4.0000
0	1	1	1	3	1	0	3	1	0	65	1	6.75	0	75.2000
1	0	2	2	3	1	1	3	1	0	64	1	3.75	1	16.0000
0	1	1	2	4	2	0	3	1	0	69		3.5	2	
1	1	1	2	4	2	0	3	1	0	76	1	5.25	1	1.3333
1	0	1	1	3	2	0	3	1	0	61	2	6.75	2	
0	1	2	2	4	1	1	3	2	0	38	2	2.25	2	
0	0	1	1	3	0	0	3	1	0	65	2	4	1	24.0000
0	1	2	1	3	2	0	2	2	0	49	1	3.75	1	6.0000
0	1	2	2	3	0	1	3	1	0	66		3.75	1	7.0000
1	1	2	2	4	1	0	2	1	0	74	2	2.75	1	4.0000
0	0	2	1	3	0	0	3	1	0	52	2	5	0	39.3333
0	1	2	1	4	0	1	2	2	1	65	1	5.5	1	2.0000
1	1	1	2	4	1	0	3	1	1	63	1	4.25	1	17.0000
1	1	2	1	4	1	0	2	1	1	65	1	6.25	1	5.0000
0	1	1	2	4	2	1	3	1	1	54		4.25	1	4.6667
0	0	2	1	3	0	0	2	1	1	73		6	0	22.6000
0	0	2	1	3	1	0	3	1	1	67	1	4.5	1	5.3333
0	1	2	2	4	2	0	2	1	1	76	1	6	1	9.1667
0	1	2	1	3	2	0	2	1	1	70		4.75	1	19.0000
0	1	1	1	4	1	0	2	1	1	67		6.25	1	11.0000
0	1	1	1	3	1	1	2	1	1	78	1	8.5	1	3.3333
0	1	2	1	3	2	0	2	1	1	64		7	1	6.0000
1	0	1	1	3	2	1	2	1	1	59	1	7.75	2	
0	1	2	1	4	2	0	3	1	1	68		8.25	1	1.6000
0	1	1	1	4	1	0	3	2	1	65	1	6.25	1	11.6667
0	1	2	1	3	0	0	2	1	1	57	1	6.75	0	35.4000
0	1	1	2	3	0	0	1	1	1	61	2	3.5	0	40.2000
1	1	2	1	3	1	0	2	1	1	70	1	6	1	2.0000
0	1	2	1	4	0	0	3	1	1	68	1	10.25	1	12.3333
0	1	1	2	4	1	1	2	1	1	63		1.5	1	10.0000
1	1	1	1	3	2	0	2	1	1	59	2	3.5	0	23.6667

0	1	1	1	3	1	0	3	1	1	61	1	5.5	2	
0	0	2	1	2	0	0	1	1	1	59		2.75	0	33.1333
0	1	1	1	3	1	0	2	2	1	66	2	4.5	2	
0	1	1	2	4	1	0	3	1	1	78	2	8.75	1	0.5000
0	0	2	2	3	1	1	3	1	1	60	1	4.5	1	1.2000
0	1	2	1	3	0	0	2	2	1	65	1	9.25	0	43.7333
1	1	1	2	4	2	1	3	2	1	53	2	6.25	1	1.8333
0	1	1	2	3	1	1	3	1	1	48	1	5.25	2	
1	1	2	1	4	1	0	2	2	1	63	1	10.25	2	

Supplementary Table S2. Records of 244 patients with GBC after surgery for modeling

Jaundice	Liver infiltration	Sex	Surgical Type	T	N	M	Pathological Grade	Pathological Type	Blood Loss	Age	Shape	Surgical Time	Survival time
0	1	1	2	4	2	0	2	1	0	63	1	3.5	3.0000
0	1	1	1	3	0	0	3	1	0	66	2	5.25	4.0000
0	1	2	2	4	1	1	2	1	0	60		1.75	2.0000
0	1	1	2	3	0	0	1	1	1	61	2	3.5	40.2000
0	1	2	2	4	2	0	3	1	0	36	1	3.5	1.6667
0	1	2	1	4	2	0	2	6	0	61	2	6.5	21.8667
0	1	2	1	3	2	0	2	1	1	64		7	6.0000
0	1	2	2	4	2	0	2	6	0	78	1	4.5	3.0000
0	1	1	2	3	1	1	2	1	0	58	1	3.75	7.2333
0	0	2	1	3	0	0	2	1	0	61	1	4	54.6333
0	0	2	1	3	1	0	2	1	0	62	2	3.75	16.0000
1	1	1	2	4	2	1	3	1	0	57		3	1.0000
0	1	1	1	3	0	0	2	1	0	68	2	3.5	40.7333
0	1	2	2	4	1	0	1	1	0	73		2.75	3.0000
0	0	2	1	3	0	0	2	1	0	58	1	4	47.6333
1	1	2	2	4	1	0	3	3	0	66	2	2	3.0000
0	0	1	2	3	2	1	3	1	0	64		2.75	0.6667
1	0	2	2	3	2	0	2	1	0	58		1.5	5.0000
0	1	1	2	4	2	0	3	3	0	68	2	3	3.0000
0	1	2	1	3	2	0	3	1	0	60		2.75	0.6667
0	1	2	1	4	0	1	2	6	1	65	1	5.5	2.0000
0	1	1	1	3	1	0	3	1	0	65	1	6.75	75.2000
0	1	1	2	4	2	0	1	1	0	68		4.75	5.0000
0	1	2	2	4	2	1	3	1	0	61		1.25	0.5000
1	0	2	1	3	0	0	3	2	0	64	2	3.75	4.8333
1	0	2	2	3	2	1	2	1	0	61	2	3	0.6667
1	1	2	2	4	1	0	3	1	0	53	1	5	9.0000
0	1	2	2	4	2	1	3	1	0	56	1	2	1.0000
0	0	2	2	3	2	0	2	1	0	76	1	1.75	7.0000
0	0	2	2	3	2	0	2	1	0	53	1	2	4.3333
0	0	2	2	3	0	0	1	1	0	57		1.25	69.8667
1	1	2	2	4	1	0	3	4	0	86	2	2.25	2.0000
0	0	2	1	3	2	0	3	1	0	56		4.5	5.3333
0	0	1	1	3	0	0	3	1	0	41	1	5.25	28.5000

0	1	2	2	3	1	1	2	1	0	52		1.75	3.0000
0	0	1	1	3	0	0	2	1	0	69		3.5	33.3000
0	1	1	1	4	1	0	2	1	1	67		6.25	11.0000
0	0	1	1	0	0	0	1	1	0	65		2	42.8000
1	1	2	2	4	2	0	3	1	0	48	2	3.75	2.0000
0	1	2	1	3	0	0	3	4	0	58	1	3	26.5000
0	1	2	2	4	1	1	2	1	0	46		3	4.0000
0	1	2	2	4	1	0	3	1	0	58		2.5	14.0000
0	0	1	1	3	1	0	3	1	0	62	2	2.25	66.7000
1	1	1	2	4	2	1	2	1	0	65		1.5	1.0000
0	1	2	2	4	2	0	2	2	0	62		3.75	1.6667
0	0	1	1	3	0	0	2	1	0	37		3.5	28.9000
0	0	2	2	3	2	1	2	4	0	76		1	3.0000
0	0	2	1	1b	2	0	3	6	0	52	2	2.75	34.2333
0	0	2	2	3	1	0	2	1	0	64	1	2.5	5.6667
0	1	1	1	3	2	0	2	1	0	66	1	2.75	5.0000
0	0	2	2	3	2	0	2	1	0	69	1	2.25	6.0000
1	0	2	2	3	1	1	3	1	0	64	1	3.75	16.0000
1	1	2	2	4	2	0	3	1	0	63		2.5	2.0000
0	0	2	1	3	0	0	3	1	0	71	2	3	7.0000
0	1	2	2	3	2	1	3	1	0	71		2.25	2.6667
0	1	1	2	4	1	1	3	1	0	56	1	4.5	6.0000
0	1	1	2	4	1	0	3	1	0	66	1	2.25	10.0000
0	1	1	1	3	0	0	3	1	0	56	1	4.5	30.2000
1	1	2	2	4	1	0	1	1	0	68		2.25	3.0000
0	1	2	1	3	2	0	2	1	0	63	1	6	9.3333
0	0	2	1	3	1	0	2	1	0	64	1	3.25	6.0000
0	1	2	2	4	1	0	3	1	0	60		2.5	1.0000
0	1	2	2	3	2	1	2	1	0	57		0.5	2.3333
0	1	2	2	4	1	0	3	1	0	76	1	2.75	2.0000
0	1	1	1	3	1	0	2	1	0	61	1	3.25	5.3333
1	1	2	2	4	1	0	3	6	0	61	1	2.75	3.3333
0	1	2	1	3	0	1	2	1	0	74	1	2.5	8.0000
0	0	2	1	3	0	0	2	1	0	69	2	1.75	42.7000
0	0	2	1	3	0	0	1	1	0	64	2	2.25	57.8000
1	1	2	2	4	1	0	2	1	0	74	2	2.75	4.0000
0	1	2	1	3	0	0	2	5	1	65	1	9.25	43.7333
0	0	1	1	3	0	0	3	1	0	65	2	4	24.0000
1	0	2	2	3	2	0	3	1	0	66	1	1.25	1.6667
0	1	1	2	3	2	0	2	2	0	65	1	1	7.0000
0	1	2	2	3	0	0	2	1	0	71	1	2	7.3333
0	0	1	1	3	0	0	2	1	0	67	2	2.25	29.9667
1	0	2	1	3	0	0	3	1	0	64	1	4.25	42.1333
0	0	2	2	2	0	0	2	1	0	75	1	2.25	3.0000
0	0	2	1	3	2	0	2	1	0	63	1	2.75	1.0000
0	1	2	2	4	1	1	3	1	0	50		1.25	1.3333
0	0	2	2	3	1	1	1	1	0	73		1.75	10.0000
0	1	1	1	3	2	0	2	1	0	64		7	5.0000

1	1	2	2	4	0	0	1	2	0	49	2	2.25	9.0000
0	0	2	1	3	0	0	2	1	0	55		3.5	36.8333
0	1	1	2	4	2	1	3	1	1	54		4.25	4.6667
0	1	1	2	4	2	0	3	6	0	51	1	2	1.6667
0	0	1	2	3	2	0	2	1	0	75	1	2.75	2.5000
1	1	1	2	4	1	1	3	1	0	49	1	2	0.0333
0	0	2	2	3	0	0	2	1	0	69		1.75	23.3000
0	0	2	1	3	0	0	2	1	0	68	2	2.75	50.3333
0	0	2	1	3	0	0	2	1	0	59	2	5.25	48.6333
0	1	2	2	4	2	0	2	1	1	76	1	6	9.1667
0	1	1	2	3	1	1	3	1	0	61		1.25	1.2000
0	0	2	1	3	0	0	3	1	0	49		4.25	23.4333
0	1	2	2	4	1	1	2	6	0	61		2.75	2.0000
0	0	2	2	3	2	0	2	1	0	69	1	2	12.1667
0	0	2	1	3	1	0	1	1	0	75		2.75	48.0000
0	1	2	2	4	1	1	3	1	0	70	1	4.5	1.0000
0	1	1	1	3	1	0	3	1	0	64		5	7.0000
0	0	2	1	3	0	0	2	1	0	62		2.75	42.6000
0	0	2	2	3	2	0	3	1	0	60	2	2.25	4.3333
0	1	2	2	4	2	1	3	1	0	60		1.5	1.3333
1	1	2	2	4	1	0	3	1	0	67	1	3.25	2.0000
0	1	1	2	3	2	0	3	1	0	50		3	8.0000
0	0	2	2	3	0	0	2	1	0	67	2	3.25	64.6667
0	1	1	1	4	1	0	3	6	1	65	1	6.25	11.6667
0	1	2	2	4	1	0	3	1	0	70		2	2.6667
0	0	1	2	3	2	1	3	1	0	62	1	1.75	1.0000
0	1	2	1	4	2	0	2	1	0	72		2.25	3.0000
0	0	2	2	3	1	0	3	1	0	60	1	2.25	14.0000
0	0	2	1	3	0	0	1	1	0	56	1	4.25	51.4333
1	1	2	1	3	1	0	2	1	1	70	1	6	2.0000
0	1	1	1	4	1	0	2	1	0	37	1	5.25	32.0000
0	1	1	2	4	1	1	3	2	0	78		2	1.0000
0	0	2	1	3	0	0	1	1	0	65	1	1.5	55.2333
0	0	2	1	3	0	0	2	1	0	44	2	4	56.6667
1	1	2	2	4	1	0	2	1	0	58		3	12.1667
0	1	1	1	3	1	1	2	1	1	78	1	8.5	3.3333
0	0	1	2	3	1	0	2	1	0	71	1	2	5.0000
0	0	2	1	1b	0	0	1	1	0	76		2.5	24.6333
0	0	1	2	3	1	1	3	1	0	83	2	2.25	2.5000
0	0	2	1	3	0	0	3	1	0	59		4.5	64.1333
0	1	2	2	4	1	1	3	5	0	51	1	4.25	3.0000
1	1	2	2	4	2	0	2	1	0	70	1	3	30.0667
0	0	2	1	3	1	0	3	1	0	39		3	4.0000
0	0	2	2	3	2	0	2	5	0	59	1	2.5	5.6667
0	0	1	1	3	0	0	2	1	0	65	1	2.5	51.0000
0	0	2	1	3	0	0	3	1	0	73	2	2.25	28.6667
1	1	2	2	4	1	0	3	1	0	66		2.25	2.0000
0	1	2	2	4	2	0	3	3	0	58	2	2.5	3.0000

0	0	2	1	3	2	0	3	1	0	49	2	3.25	2.0000
0	0	2	1	3	1	0	3	1	0	71	1	4.75	7.0000
1	1	1	2	4	2	1	2	1	0	66		4.5	3.0000
0	1	2	1	3	2	0	2	1	0	57	1	5.75	7.6667
1	1	2	2	3	1	1	2	1	0	46		3.75	5.5000
1	0	2	2	3	2	1	2	1	0	81		3	4.0000
0	0	2	2	3	2	0	3	1	0	73	1	3.25	7.0000
0	0	1	1	3	0	0	2	1	0	65	1	2.5	27.5333
1	1	1	2	4	2	0	3	1	0	76	1	5.25	1.3333
1	0	1	2	3	1	0	2	1	0	83		4.75	5.4667
0	1	2	2	3	2	1	3	1	0	69		1.5	1.0000
0	0	2	1	3	1	0	3	1	0	67	2	1.75	8.0000
0	0	1	1	3	0	0	2	1	0	74	2	2.25	63.4333
0	0	2	2	3	1	1	3	1	1	60	1	4.5	1.2000
0	1	2	1	4	2	0	3	1	1	68		8.25	1.6000
0	0	2	1	3	0	0	3	4	0	46		2.75	23.2000
0	1	2	2	3	2	0	3	1	0	51	1	3.5	4.3333
0	0	2	1	3	1	0	3	1	0	70	1	1.5	6.9000
1	1	2	2	4	2	0	3	1	0	77	1	3	0.9000
0	1	1	1	3	1	0	3	6	0	73	1	3.5	3.0000
0	1	1	2	4	1	0	3	1	0	66	1	1.25	3.6667
1	0	2	2	3	2	1	3	1	0	65	1	3	3.0000
0	0	2	1	1a	0	0	1	1	0	65		2.75	25.4000
0	1	1	2	4	1	1	2	1	0	62	2	3	2.6667
1	1	2	2	4	1	0	2	1	0	68	1	5.5	2.0000
0	0	2	1	3	2	0	3	1	0	62		2	6.0000
0	1	1	2	4	1	0	2	2	0	65		4	3.3333
0	1	2	2	4	2	1	2	5	0	65	1	1	3.0667
0	1	1	2	3	0	0	2	1	0	51	1	5.75	23.3333
0	0	2	1	3	1	0	1	4	0	52	2	2.5	31.8000
1	1	2	2	3	2	1	3	1	0	44		3	1.6667
0	0	2	1	3	0	0	2	1	0	77	2	4.75	45.7667
1	1	1	2	3	1	1	3	1	0	69		3.25	12.0000
1	1	2	2	4	2	0	3	1	0	49	1	3	2.6667
0	0	2	2	3	1	1	3	1	0	65		1.5	2.0000
1	0	2	2	3	1	1	3	1	0	72	2	2.75	1.3333
0	1	2	2	3	2	1	3	3	0	63	1	2.5	3.5000
0	0	2	2	1a	0	0	1	1	0	81		3	30.1667
0	1	2	2	3	2	1	3	1	0	54		1.5	1.0000
0	0	2	1	0	0	0	1	1	0	43	2	2.25	54.1000
1	1	1	2	4	2	1	3	1	0	58		0.25	1.0000
0	0	2	1	3	1	0	3	1	0	52	1	4	33.6333
0	0	2	1	1b	0	0	2	1	0	75	1	2.5	51.0333
0	0	1	2	3	0	0	2	1	0	74	2	1.5	13.0000
0	0	2	2	3	0	0	2	1	0	79	1	1	62.5000
0	1	2	2	3	1	1	3	1	0	60		0.5	0.2333
0	1	1	2	4	1	1	3	1	0	71		2.25	1.0000
0	1	2	2	4	1	0	3	1	0	65		3.25	2.0000

0	0	1	2	3	2	0	2	1	0	65	1	4	2.0000
0	1	2	2	4	2	1	3	1	0	57		2.75	1.3333
0	1	2	2	3	2	0	2	1	0	48	1	4	6.0000
1	1	2	2	4	1	1	2	2	0	71	1	2.75	1.6667
0	0	2	1	3	0	0	3	1	0	66	1	4	41.3000
1	1	1	2	4	1	0	3	1	0	63	1	3.5	9.0000
0	1	1	2	4	2	0	3	1	0	72		2.75	3.0000
0	1	2	2	3	2	1	3	1	0	71		0.75	0.5000
0	0	2	1	1b	0	0	2	1	0	61	1	2.75	59.7333
0	1	1	2	4	1	0	2	1	0	58		2.75	5.3333
0	0	1	1	3	1	0	3	1	0	75	2	4.5	44.9000
0	1	1	2	4	2	1	3	1	0	59		3	1.3333
0	0	2	1	3	1	0	3	1	0	44	2	5.5	24.0000
1	0	2	2	3	0	0	1	1	0	68		2.5	52.3667
0	0	2	1	3	2	0	3	1	0	53	2	3.5	12.1667
0	1	2	1	3	0	0	3	1	0	39		3.5	66.4667
1	0	2	2	3	2	1	2	1	0	66	1	1.25	2.0000
1	1	2	2	4	1	0	2	1	0	83		4.25	0.6667
0	1	2	2	3	2	1	3	1	0	59	2	5.75	1.0000
0	1	1	2	4	1	0	2	1	0	51	1	3	6.6667
1	0	2	2	3	0	0	1	1	0	78	1	2.25	63.5000
0	1	2	2	3	0	1	3	1	0	66		3.75	7.0000
0	1	2	2	3	1	1	3	6	0	65	2	3.75	2.0000
0	1	2	2	3	2	1	3	1	0	59		1.5	2.3333
0	0	2	1	3	0	0	2	1	0	46	1	2	57.5000
0	0	2	2	3	0	0	3	1	0	56	2	3	79.1000
1	0	2	2	3	0	0	2	1	0	76	2	2	57.1333
1	0	1	1	3	0	0	1	4	0	61		1.75	75.2000
1	1	1	1	3	1	0	2	1	0	54	1	3.5	39.7667
0	1	1	2	4	2	1	3	6	0	50		2.5	1.5000
0	1	1	2	4	1	0	3	1	1	78	2	8.75	0.5000
0	1	1	2	4	1	1	2	1	0	61	1	1.5	2.0000
0	1	2	1	4	0	0	3	1	1	68	1	10.25	12.3333
0	1	2	2	4	1	0	3	1	0	42		2	6.0000
0	1	2	2	4	2	0	3	3	0	38		2.5	4.3333
1	0	2	2	3	0	0	1	2	0	82	2	2	5.0000
0	1	2	2	4	1	0	2	1	0	86	1	2.25	4.0000
0	0	2	1	3	0	0	2	1	0	71	2	3	45.5667
0	0	2	2	3	2	1	1	1	0	61	1	3.75	3.0000
1	1	2	2	3	2	1	3	1	0	60		1.75	1.0000
1	1	2	2	4	1	0	3	1	0	72		4	2.0000
1	0	2	1	3	1	0	3	1	0	78	1	5	7.0000
1	1	2	2	4	1	0	3	1	0	48	1	2	6.3333
0	1	1	2	4	1	0	2	1	0	68	1	2.75	4.6667
1	1	1	2	3	1	1	2	1	0	39	2	2	5.0000
0	0	2	1	2	0	0	3	3	0	49		3	23.3333
0	0	1	2	3	0	0	2	1	0	59	2	3.25	80.4667
0	1	1	2	4	2	0	2	1	0	84	1	2.5	1.1667

0	0	2	1	3	1	0	2	1	0	63		3	7.0000
0	1	2	1	3	2	0	2	1	0	60	1	5	1.0000
0	1	2	2	4	1	1	2	1	0	67		0.5	1.6667
0	1	2	2	4	1	0	2	5	0	71	1	3.75	2.0000
0	0	2	1	2	0	0	1	1	1	59		2.75	33.1333
0	1	2	2	4	2	1	3	1	0	56		1	1.5000
0	0	2	1	3	0	0	1	1	0	75	1	3.5	77.8000
0	1	2	2	3	2	0	2	1	0	50	1	4	6.0000
0	1	1	2	3	1	1	1	4	0	75		3	2.0000
1	1	2	2	4	1	1	2	1	0	81		3	2.3333
0	1	1	2	4	2	0	2	1	0	66		2	2.3333
0	0	2	1	3	0	0	3	1	0	42	2	5.75	51.4667
0	0	1	2	3	0	0	2	1	0	51		1.75	77.1000
1	1	1	2	3	2	0	3	5	0	78	1	4.25	0.6000
0	0	2	2	1b	0	0	1	1	0	60	2	3	63.3333
1	0	2	2	3	2	0	3	1	0	65	1	2.5	2.0000
1	1	1	2	4	2	1	3	3	1	53	2	6.25	1.8333
0	1	2	1	4	0	0	3	1	0	45	1	6.75	3.3000

Supplementary Table S3. Records of 122 patients with GBC after surgery for testing

Jaundice	Liver infiltration	Sex	Surgical Type	T	N	M	Pathological Grade	Pathological Type	Blood Loss	Age	Shape	Surgical Time	Survival time
0	0	1	1	3	0	0	3	1	0	52		3.25	41.5333
0	0	1	1	3	1	0	1	1	0	62	1	6	29.8667
0	1	2	1	3	0	0	2	1	1	57	1	6.75	35.4000
0	0	1	2	3	2	0	2	1	0	56		2.5	3.6667
0	0	2	1	3	1	0	3	1	0	51		2.5	67.4000
0	1	2	1	3	2	0	2	6	0	49	1	3.75	6.0000
0	1	2	2	4	2	1	2	1	0	65		3	1.1667
0	0	2	1	3	1	0	2	1	0	61		4.5	22.0667
0	1	1	2	4	2	1	3	1	0	51		1	2.0000
0	0	2	1	3	0	0	1	1	0	62		4.75	44.3333
0	1	2	2	4	2	1	3	1	0	50		2	1.3333
1	1	1	2	4	2	1	2	1	0	68		1.75	6.1667
0	0	1	2	3	0	0	2	1	0	82	1	1.25	37.2333
0	0	2	1	3	0	0	2	1	0	65	2	1.75	78.4000
0	0	2	2	3	0	0	2	1	0	68	1	1.75	52.9333
0	0	2	2	3	1	1	3	1	0	52	1	2.75	6.6667
0	0	1	1	3	0	0	2	1	0	48		3.25	30.1333
0	0	2	2	3	1	0	1	1	0	84	1	3.25	15.0000
0	1	1	2	3	1	1	3	1	0	60		3	18.8333
1	1	2	2	4	1	0	2	1	0	55	1	4	4.0000
0	1	2	2	4	1	1	2	1	0	72	1	4	3.0000
1	0	1	2	3	2	0	2	1	0	45		2.25	1.0000
1	1	1	2	4	1	0	2	1	0	51		3	8.0000
0	1	2	2	4	2	1	1	4	0	75		1.25	0.4333
0	1	2	1	3	1	1	2	1	0	66		2.5	1.3333

0	1	2	2	4	1	1	2	1	0	71	1	4.5	2.3333
0	1	2	2	4	2	1	3	1	0	67		1	1.0000
0	0	2	2	3	2	1	3	1	0	55		4	2.3333
1	1	2	2	4	1	0	2	1	0	75	2	2	2.6667
0	0	2	1	3	0	0	1	1	0	63		2.5	37.7667
0	1	2	1	3	0	0	1	2	0	61	2	2.5	55.4667
0	0	2	1	3	0	0	3	1	0	52	2	5	39.3333
1	1	2	1	4	1	0	2	1	0	58		2	7.5333
0	1	1	2	3	2	0	2	1	0	63	1	4	3.0000
1	1	2	2	4	2	0	2	1	0	49	2	2.75	6.0000
0	0	1	1	1b	0	0	2	1	0	46		3	32.3000
1	1	1	2	4	1	0	3	1	1	63	1	4.25	17.0000
0	0	1	2	3	1	0	3	1	0	71	1	0.5	9.0000
1	0	2	1	3	1	0	3	1	0	74	1	5.5	12.5000
1	0	2	1	3	1	0	3	1	0	56		2.5	1.3333
1	1	2	1	3	2	0	2	1	1	59	2	3.5	23.6667
0	1	2	2	4	2	1	3	1	0	57	1	3	51.4667
0	1	1	2	3	0	0	2	1	0	54	1	2.25	20.0000
1	0	1	1	2	0	0	1	1	0	54		4	38.1333
0	1	2	2	4	1	1	2	1	0	59	1	2.75	3.0000
1	1	1	1	4	1	0	2	1	1	65	1	6.25	5.0000
0	0	2	1	3	0	0	1	1	0	64	1	3	47.8000
0	1	2	2	4	1	1	3	1	0	41		3.25	3.0000
0	1	2	2	4	1	0	3	6	0	74		2.5	5.0000
1	0	1	1	3	1	0	3	5	0	51	1	5.25	4.0000
0	0	2	1	3	1	0	3	1	0	39	2	3.5	2.0000
0	0	2	1	3	0	0	1	1	0	61	2	3	3.6667
1	1	2	2	4	1	0	2	1	0	51		3.5	1.0000
0	0	2	1	3	2	0	3	1	0	63	1	4.75	5.6667
1	1	2	1	3	1	0	2	1	0	65		6	39.6000
0	1	1	2	3	2	0	3	5	0	62	1	3.25	10.0000
0	1	1	2	4	1	1	3	1	0	62	1	4.75	1.6667
0	1	1	1	3	2	0	3	1	0	38	1	5.5	11.6667
0	1	2	2	4	2	0	3	1	0	70		1.5	2.3333
1	1	2	1	3	2	0	2	1	0	60	1	5.25	9.0000
0	0	2	2	3	2	1	3	1	0	68	1	2.5	1.3333
0	1	2	1	4	1	0	2	6	0	65		5.25	12.1667
0	0	2	1	3	0	0	2	1	0	59		4	38.3333
1	0	2	1	3	0	0	2	1	0	55	2	3	63.5000
0	1	1	1	3	0	0	3	6	0	68	1	4.5	2.0000
0	0	2	1	3	1	0	3	1	1	67	1	4.5	5.3333
0	0	2	2	3	1	1	3	1	0	55		2.5	3.0000
0	0	2	2	3	1	1	2	1	0	48	2	4	5.0000
0	0	2	2	3	1	1	1	1	0	55		6	7.0000
0	0	2	2	3	2	0	3	1	0	53	2	3.5	2.0000
1	0	2	1	1b	0	0	1	1	0	83	2	4.75	31.3333
1	1	1	1	3	0	0	2	1	0	64	1	6.75	15.0000
0	0	2	1	3	0	0	2	1	0	55	1	5.75	43.2333

0	1	1	2	3	1	1	3	1	0	55	1	3.25	1.6667
0	1	2	2	3	2	1	3	3	0	59	2	2.5	1.0000
0	0	1	2	3	1	1	3	1	0	76	1	2.25	1.0000
1	0	2	1	3	0	0	3	1	0	71		2.75	23.1667
0	1	2	1	3	2	0	2	1	1	70		4.75	19.0000
0	0	2	2	3	0	0	3	1	0	71		1.25	74.9333
0	1	2	2	4	1	1	2	1	1	63		1.5	10.0000
1	1	2	2	4	1	0	2	1	0	60		2	8.0000
0	1	1	2	4	2	1	3	1	0	75		5	1.0000
0	0	2	1	3	1	0	3	1	0	54	1	4.5	4.0000
0	1	2	2	4	2	1	3	1	0	79	1	5.25	1.3333
0	0	1	1	3	0	0	2	1	0	62	1	4	11.0000
1	1	1	2	4	1	0	3	1	0	79		2.25	3.0000
0	0	1	1	3	0	0	2	1	1	73		6	22.6000
0	1	1	2	3	2	1	2	5	0	45	2	2.75	4.0000
1	1	2	2	4	1	0	3	1	0	46		2.75	29.1333
0	1	2	2	3	2	1	3	1	0	64	1	2.5	3.0000
0	0	2	1	3	0	0	2	1	0	29	2	1.75	54.7333
0	0	2	1	3	0	0	2	1	0	72	1	3.5	20.0000
1	1	1	2	4	2	0	3	1	0	66		4	1.0000
0	1	2	2	3	1	1	1	1	0	61	1	2.25	0.3000
0	1	2	1	3	2	0	3	3	0	65	1	2.5	5.6667
0	1	2	2	4	2	0	3	1	0	66		2	2.0000
0	1	2	2	4	2	1	2	1	0	55	1	2.75	2.6667
0	0	2	1	3	0	0	1	1	0	50		2.5	22.0000
1	1	1	2	4	1	1	2	1	0	62		2.75	2.0000
0	0	2	2	3	1	0	3	1	0	67		2.5	3.0000
0	0	2	2	1b	0	0	2	1	0	63		2.75	74.0667
1	0	2	2	3	2	0	2	5	0	78		3	10.0000
0	1	2	2	4	0	0	3	1	0	65	1	5	38.0000
0	0	1	1	1a	0	0	3	1	0	52	1	6.25	39.1667
0	1	2	2	4	2	1	3	1	0	70	1	4.75	1.0000
1	1	1	2	4	2	0	2	1	0	54	1	2.5	5.2000
1	1	2	2	4	1	0	3	1	0	64	1	4.75	1.0000
1	0	1	2	3	2	0	3	5	0	43	1	2.25	10.0000
1	1	2	2	4	1	0	3	1	0	51		2.75	2.0000
1	0	2	2	3	2	1	2	1	0	41		1.75	3.0000
0	0	2	2	3	2	0	2	1	0	68	2	1.5	10.0000
0	0	2	1	3	0	0	2	1	0	78	1	3.25	32.3000
0	1	1	2	4	2	0	3	6	0	61	2	3.25	1.6667
1	0	1	1	3	0	0	1	1	0	60	2	1.25	65.7000
0	1	2	2	4	1	0	2	6	0	47	1	4	7.0000
1	0	2	1	0	0	0	1	1	0	78		2.25	26.8667
0	0	2	1	2	0	0	2	1	0	66		3.5	69.6667
0	1	1	2	4	1	0	2	1	0	49	1	4.5	14.0000
0	0	1	2	3	2	1	3	1	0	67	1	2.75	1.0000
0	1	2	1	3	1	0	2	6	0	55	1	5.25	10.0000
1	1	1	2	4	1	0	3	1	0	68	1	5	5.0000

0	1	2	2	4	1	1	3	1	0	64		3.5	1.3333
